# Posttranslational hypusination of the eukaryotic translation initiation factor-5A regulates *Fusarium graminearum* virulence

**DOI:** 10.1038/srep24698

**Published:** 2016-04-21

**Authors:** Ana Lilia Martinez-Rocha, Mayada Woriedh, Jan Chemnitz, Peter Willingmann, Cathrin Kröger, Birgit Hadeler, Joachim Hauber, Wilhelm Schäfer

**Affiliations:** 1 University of Hamburg, Biocenter Klein Flottbek, Molecular Phytopathology and Genetics, Hamburg, D-22609, Germany; 2 Heinrich Pette Institute, Leibniz Institute for Experimental Virology, Department Antiviral Strategies, Hamburg, D-20251, Germany; 3 German Center for Infection Research (DZIF), partner site Hamburg, Hamburg, Germany

## Abstract

Activation of eukaryotic translation initiation factor eIF5A requires a posttranslational modification, forming the unique amino acid hypusine. This activation is mediated by two enzymes, deoxyhypusine synthase, DHS, and deoxyhypusine hydroxylase, DOHH. The impact of this enzymatic complex on the life cycle of a fungal pathogen is unknown. Plant pathogenic ascomycetes possess a single copy of the eIF5A activated by hypusination. We evaluated the importance of imbalances in eIF5A hypusination in *Fusarium graminearum*, a devastating fungal pathogen of cereals. Overexpression of *DHS* leads to increased virulence in wheat, elevated production of the mycotoxin deoxynivalenol, more infection structures, faster wheat tissue invasion in plants and increases vegetatively produced conidia. In contrast, overexpression of *DOHH* completely prevents infection structure formation, pathogenicity in wheat and maize, leads to overproduction of ROS, reduced DON production and increased sexual reproduction. Simultaneous overexpression of both genes restores wild type-like phenotypes. Analysis of eIF5A posttranslational modification displayed strongly increased hypusinated eIF5A in *DOHH* overexpression mutant in comparison to wild type, and the *DHS* overexpression mutants. These are the first results pointing to different functions of differently modified eIF5A.

The eukaryotic translation initiation factor 5A (eIF5A), a small acidic protein found in eukaryotes and archea but not in eubacteria, was proposed to function during translation initiation as a nucleo-cytoplasmic shuttle for a subset of mRNAs necessary during cell cycle progression from G1 to S phase^1^,^2^. Recent studies implicated eIF5A in stimulating ribosome peptidyl transferase activity to promote translation elongation, particularly of proteins containing poly-proline sequences^3^. Temperature-sensitive mutants of eIF5A in yeast suggested a direct or indirect role of eIF5A in cell cycle progression, cell wall integrity, mRNA decay, actin polarization, and anti-apoptotic protection^4^,^5^,^6^. Activation of eIF5A requires a posttranslational modification forming the unique amino acid hypusine. This posttranslational modification, only found in the eIF5A protein, is mediated by two enzymes, deoxyhypusine synthase (DHS) and deoxyhypusine hydroxylase (DOHH). In the first step, DHS cleaves a 4-aminobutyl moiety from polyamine spermidine and adds it to a specific lysine in eIF5A to create the deoxyhypusine intermediate Dhp-eIF5A. This intermediate serves as a substrate for the second enzymatic step where DOHH hydroxylates the 4-amonibutyl moiety to create the active form of eIF5A containing hypusine (Hyp-eIF5A)^7^. Gene disruption and/or mutations of eIF5A, DHS and DOHH in yeast, as well as silencing or inhibiting gene expression in higher eukaryotes, revealed the essential function of hypusine-eIF5A in cell proliferation^8^,^9^. The high specificity of DHS and DOHH biosynthetic enzymes points to the importance of this unique posttranslational modification and its potential as a new antiproliferative target^10^,^11^.

We recently described the essential role played by DHS in the virulence of *Fusarium graminearum* (teleomorph *Gibberella zeae*), the major causal agent of the devastating diseases Fusarium Head Blight (FHB) in small-grain cereals and cob rot in maize^12^. These recurrent diseases cause losses in yield, contaminate products with mycotoxins and reduce grain quality^13^,^14^,^15^,^16^. To develop novel compounds for long-term control of *F. graminearum*, it is important to understand the molecular basis of the pathogen’s life cycle^14^. A recent example of using novel compounds to reduce disease incidence in both wheat and maize was the external application of CNI-1493, a compound that inhibits fungal DHS activity without affecting grain development. This specific inhibitor of DHS confirmed that this protein is essential in *F. graminearum*^12^. In this study we evaluated the importance of the two enzymes which activate eIF5A post-translationally during the life cycle of *F. graminearum*.

## Results

### Hypusine biosynthetic genes are essential for cell survival

We identified and characterized *EIF5A* and *DOHH* genes to determine the importance of eIF5A hypusination in *F. graminearum*. The open reading frame (ORF) of *EIF5A* comprises 888 base pair (bp) interrupted by three introns of 242, 99 and 55 bp, encoding a protein of 165 amino acids (aa) with a predicted molecular mass of 18 kDa and an isoelectric point of 4.7. Alignment of the eIF5A1 amino acid sequence from *F. graminearum* with different species reveals high similarity, ranging from 98.8% with *Fusarium oxysporum* eIF5A1 to 54.7% with *Triticum aestivum* eIF5A1. The hypusine domain comprising 10 amino acids is identical in all eIF5A protein sequences. The second lysine in the hypusine domain is described as the site of hypusination (lysine 56 in *F. graminearum*, Supplementary Fig. S1A)^17^.

The coding sequence of the *DOHH* gene comprises 1062 bp interrupted by one intron of 48 bp, encoding a protein of 338 aa with a predicted molecular mass of 36 kDa and an isoelectric point of 4.5. Molecular mass and isoelectric point of eIF5A and DOHH were predicted using the *Scansite Molecular Weight & Isoelectric Point Calculator* program^18^. Alignment of the DOHH amino acid sequence from *F. graminearum* revealed high similarity among the DOHH proteins and the conserved histidine-glutamic acid HE motifs (from 91.5% with *F. oxysporum* DOHH to 45.5% with *Homo sapiens* DOHH, Supplementary Fig. S1B).

To determine whether the *DOHH* gene is essential for cell viability, we disrupted the gene by replacing the entire ORF with a hygromycin selection marker cassette. Five independent transformations resulted in a low number of ectopic transformants. PCR analysis confirmed the lack of homologous integrations, indicating that the disrupted *DOHH* genotype is not viable (Supplementary Fig. S2).

### Overexpression of *DHS* and/or *DOHH* disturbed conidiation, germination and perithecia production

To assess their importance in eIF5A hypusination, targeted *DHS* or *DOHH* overexpressing mutants (DHSoex, DOHHoex) were generated by single crossover event inserting the linearized vectors pMW-DHS or pMW-DOHH (Supplementary Table S1, Supplementary Fig. S3A,B). Southern blots confirmed the replacement of the endogenous *DHS* and *DOHH* genes by homologous recombination with the PgpdA-DHS or Pgpd1-DOHH alleles, excluding any possibility of mutagenic effects by ectopic integration (Supplementary Fig. S3C). In addition, a double overexpressing mutant (DHSoex/DOHHoex) was generated by transforming the DHSoex overexpressing mutant with the pMW-DOHH construct. The transformants showing homologous recombination were used in further analysis. We confirmed overexpression of the *DHS* and *DOHH* genes by reverse transcription-quantitative polymerase chain reaction (RT-qPCR) performed on RNA extracted from mycelia of the overexpressing mutants and the wild type strain. In the single overexpressing mutants, expression levels of *DHS* or *DOHH* were 60- or 65- fold higher than in the wild type strain. In the double overexpressing mutant expression levels of *DHS* and *DOHH* were up-regulated 30- and 25-fold compared to the wild type. Furthermore, expression levels of *EIF5A* in the overexpressing mutants were unaffected as well as the levels of *DOHH* in the DHSoex mutant or levels of *DHS* in the DOHHoex mutant (Supplementary Fig. S3D).

High levels of *DHS* or *DOHH* proteins in the respective overexpressing mutants were confirmed by Western blots using antibodies against human DHS or *F. graminearum* DOHH. In the DHSoex and double overexpressing mutants DHS protein levels were 55% and 45% more than in the wild type or DOHHoex mutant. DOHH protein levels were 31% and 26% more than in the wild type or DHSoex mutant (Supplementary Fig. S4).

To characterize DHSoex and DOHHoex mutants, we first assessed two key aspects of the pathogen’s life cycle, conidia production from vegetative asexual growth and perithecia production from sexual reproduction, as well as assessing the respective germination of conidia and ascospores. Conidia morphology of the overexpressing mutants did not differ from the wild type strain. However, the DHSoex mutant produced 30% more conidia, the DOHHoex produced 54% less conidia and the double overexpressing mutant showed levels of conidiation similar to the wild type. The germination rate (%) was determined by counting bipolar, unipolar, or non-germinated conidia. The DHSoex and DHSoex/DOHHoex mutants showed wild type germination rates. However, germination in the DOHHoex mutant was highly impaired: bipolar germination decreased, unipolar germination remained similar, and non-germination increased drastically compared to the wild type strain. Perithecia production was quantified by counting the number of perithecia per square cm that had formed on wheat nodes. Surprisingly, the DOHHoex mutant produced 5 times more perithecia than the wild type, while wild type, DHSoex and DHSoex/DOHoex strains formed similar numbers of perithecia. In addition, when we determined the viability of discharged mature ascospores by germinating them in water, all strains produced similar numbers of viable ascospores (Supplementary Fig. S5).

### *DHS* and *DOHH* overexpression alters stress responses and ROS production

To determine whether the overexpressing mutants have wild type-like responses to different stresses, they were subjected to phenotypic analysis on CM plates containing components such as sodium chloride (1.5 M NaCl) for osmotic stress, hydrogen peroxide (20 mM H_2_O_2_) for oxidative stress and calcofluor white (0.5 mM CFW) for cell wall stress. The DHSoex mutant was more sensitive to oxidative stress than the wild type strain, and the DOHHoex mutant was highly sensitive to osmotic stress and cell wall stress. The phenotype of the double overexpressing mutant was similar to the wild type. In addition, the fungicides tebuconazole, which impairs ergosterol synthesis, and azoxystrobin, which prevents ATP generation in the mitochondria, were added to complete media to analyze fungicide resistance. Whereas the DHSoex mutant was more sensitive to tebuconazole, the DOHHoex mutant was more resistant to both tebuconazole and azoxystrobin than the wild type strain. The double overexpressing mutant’s phenotype was similar to the wild type (Supplementary Fig. S6 and Supplementary Table S2).

**Figure 1:**
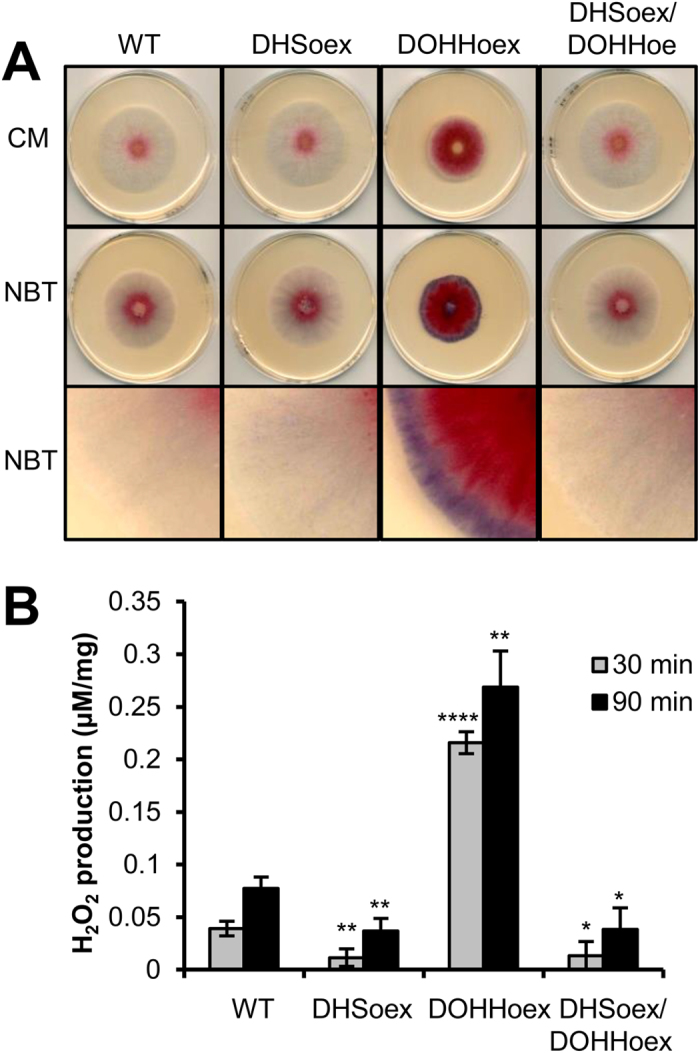
*DOHH* overexpression induces ROS production in culture. The DOHHoex mutant produces more superoxide (O_2_^−^) and hydrogen peroxide (H_2_O_2_) than the wild type strain or DHSoex and DHSoex/DOHHoex mutants. (**A**) Production of superoxide was visualized after growing the wild type strain and the overexpressing mutants on CM plates for 3 days and subsequent staining with nitroblue tetrazolium (0.2% NBT). The images are representative of 5 independent experiments. (**B**) 20 mg of mycelia grown for 3 days in liquid CM were used to determine hydrogen peroxide (H_2_O_2_) production using the Amplex^®^ Red Hydrogen Peroxide/Peroxidase Assay Kit (Invitrogen). Error bars indicate standard deviations calculated from data per triplicate samples and are representative of three independent experiments. Significance with respect to wild type: *p < 0.05, **p < 0.01, ****p < 0.0001 (calculated with ANOVA-Bonferroni-Holm).

Based on the phenotypes revealed in the stress-related tests above, we compared ROS production in the overexpressing mutants and wild type strain. We qualitatively measured superoxide (O_2_^−^) by staining mycelia grown on CM plates for 3 days with nitroblue tetrazolium (0.2% NBT), which is reduced upon exposure to superoxide anions to produce a dark blue water-insoluble formazan precipitate. Overexpression of *DOHH* caused more production of superoxide radicals than in the DHSoex and DHSoex/DOHHoex mutants or wild type strain (Fig. 1A). In addition, we quantified the production of hydrogen peroxide (H_2_O_2_) in mycelia grown for 3 days in liquid CM using an AmplexRed-kit (Invitrogen). Similar to the above result, overexpression of *DOHH* caused a 3-fold over-production of H_2_O_2_. In contrast, overexpression of *DHS*, or both *DHS* and *DOHH* together produced levels of H_2_O_2_ similar to the wild type strain (Fig. 1B).

### DHSoex is hyper-virulent but DOHHoex lacks virulence towards wheat

**Figure 2:**
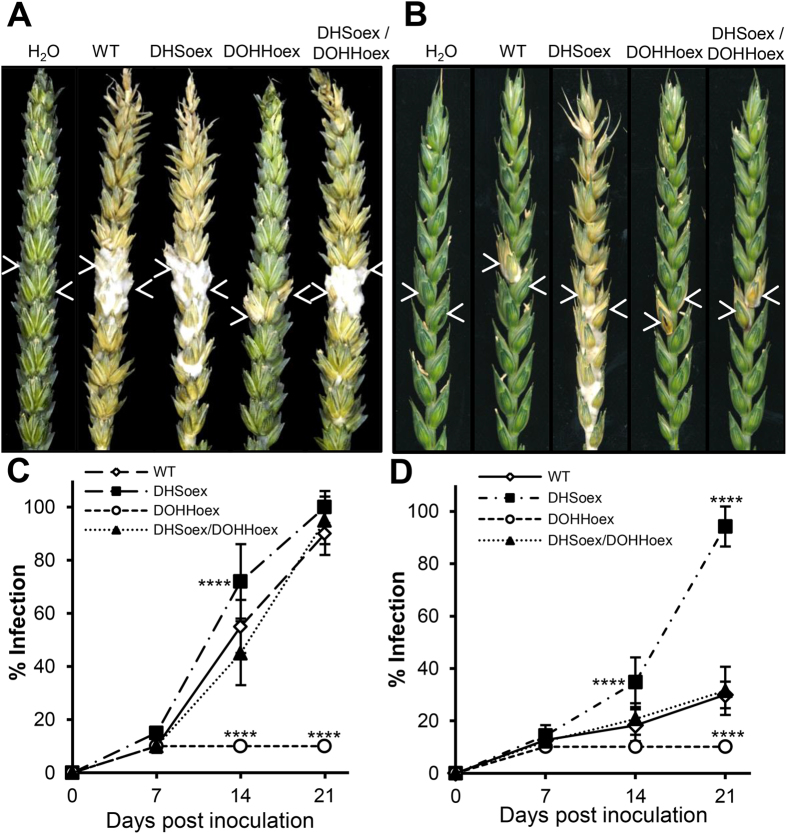
Overexpressing mutants DHSoex and DOHHoex alter pathogenesis in *F. graminearum*. Wheat spikes were inoculated with 10 μl of a suspension of 2 × 10^4^ conidia per ml. Water was used as a negative control. Percentage of infection was determined at 7, 14 and 21 dpi. Arrows indicate the point of inoculation. Pictures were taken 21 dpi. (**A**) On the susceptible cultivar Nandu, wild type (WT), DHSoex and DHS/DOHHoex strains produced a full infection in contrast to the DOHHoex mutant, which only infected the inoculated spikelets. (**B**) On the partially resistant cultivar Melissos, WT and the DOHHoex, DHSoex/DOHHoex mutants, only infected the inoculated spikelets, whereas the DHSoex mutant produced a full infection. (**C,D**) Symbols on the graphs refer to plants inoculated with the wild type (open diamonds), DHSoex (filled squares), DOHHoex (open circles) and DHSoex/DOHHoex (filled triangles). Error bars indicate standard deviations calculated from 10 spikes for each treatment and 3 independent experiments (n = 30). Significance with respect to wild type: ****p < 0.0001 (calculated with ANOVA-Bonferroni-Holm).

We explored the pathogenicity towards wheat by infecting the susceptible cultivar Nandu with the overexpressing mutants and the wild type strain, and evaluate fungal virulence at 7, 14 and 21 days post inoculation (dpi). Spikes infected with DHSoex, DHSoex/DOHHoex and the wild type strains showed full infection and bleaching at 21 dpi. In contrast, infection symptoms in spikes inoculated with the DOHHoex mutant were only visible at the inoculated spikelets. Mock inoculation with water resulted in no symptoms (Fig. 2A). To determine whether the DHSoex is indeed more virulent than the wild type, we performed experiments on the partially resistant cultivar Melissos. Infection symptoms in wheat spikes inoculated with the DHSoex mutant expanded faster and further than in spikes infected by the wild type or DHSoex/DOHHoex strains. Again, the DOHHoex mutant only produced symptoms in the inoculated spikelets (Fig. 2B). Monitoring the progression of infection indicated that the DHSoex mutant had a slightly faster infection rate than the wild type strain in the cultivar Nandu (Fig. 2C). Furthermore, the DHSoex mutant was the only one able to fully infect the partially resistant cultivar Melissos (Fig. 2D).

**Figure 3:**
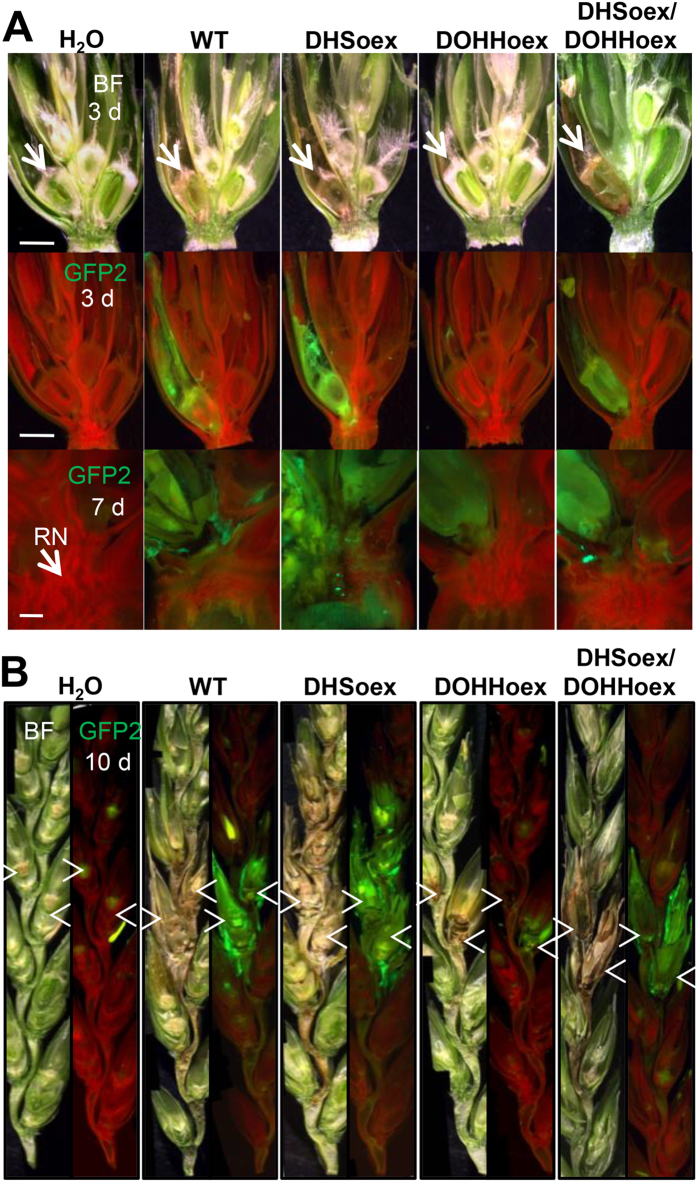
Overexpressing mutants affect infection progression of *F. graminearum* in wheat plants. Wheat plants (cv. Nandu) were infected with the wild type and overexpressing mutants carrying a constitutive EGFP reporter gene. Bright field microscopy (BF) shows browning and bleaching of the infected floret. GFP reveals colonization of the caryopsis and adjacent spikelets by the wild type, DHSoex and DHSoex/DOHHoex strains. Water was used as a negative control. The DOHHoex mutant shows minimal colonization, whereas the DHSoex shows faster colonization. **(A)** Micrographs of spikelet transversal sections at 3 dpi (3 d, scale bar = 2 mm) or 7 dpi (7 d, scale bar = 0.5 mm). RN: Rachis node. (**B**) Infection progression inside a spike at 10 dpi. Arrows mark the points of inoculation. Micrographs were taken with a fluorescence stereomicroscope (MZFLIII, Leica) and are representative of 10 infected spikes.

Infection-defective mutants are able to invade caryopses, but cannot trespass the rachis node barrier during wheat head infection^19^,^20^. Thus, to analyze wheat infection at early time points and observe progression of fungal growth inside a spike, we constitutively expressed the EGFP fluorescent protein in the overexpressing mutants and wild type strain (Supplementary Fig. S7). Analysis of infection at 3 dpi showed browning of the caryopses caused by the DHSoex, DHSoex/DOHHoex and wild type strains, but not by the DOHHoex mutant. The browning of the caryopses observed with bright field microscopy correlated with EGFP detected with the fluorescence channel, inferring that necrosis occurs at the sites of overexpressing and wild type strains infection. At 7 dpi the DHSoex/DOHHoex and wild type strains had started to infect the adjacent caryopsis. The DHSoex mutant had already colonized the adjacent caryopsis and passed the rachis node barrier. However, infection progression of the DOHHoex mutant was seen in the caryopsis, but did not trespass across the rachis node into the adjacent spikelet (Fig. 3A). By 10 dpi the wild type strain and double overexpressing mutant had fully infected the two inoculated spikelets and had started to invade the next one. The DHSoex mutant had already reached more than three adjacent spikelets, whereas the DOHHoex mutant had not been able to cross the rachis node barrier (Fig. 3B).

### DHSoex increases but DOHHoex reduces infection structures and DON mycotoxin production

Recently, we described that *F. graminearum* undergoes a morphological transition from epiphytically growing hyphae to complex infection structures during early infection steps. Structures such as lobate appresoria and infection cushions form in places where stringent contact with the plant occurs and are necessary for penetration. In addition, the *TRI5* gene, which encodes a trichodiene synthase, the first enzyme necessary to produce the mycotoxin deoxynivalenol (DON), is induced in such infection structures; however, lack of *TRI5* does not interfere with the formation of infection structures^21^.

**Figure 4:**
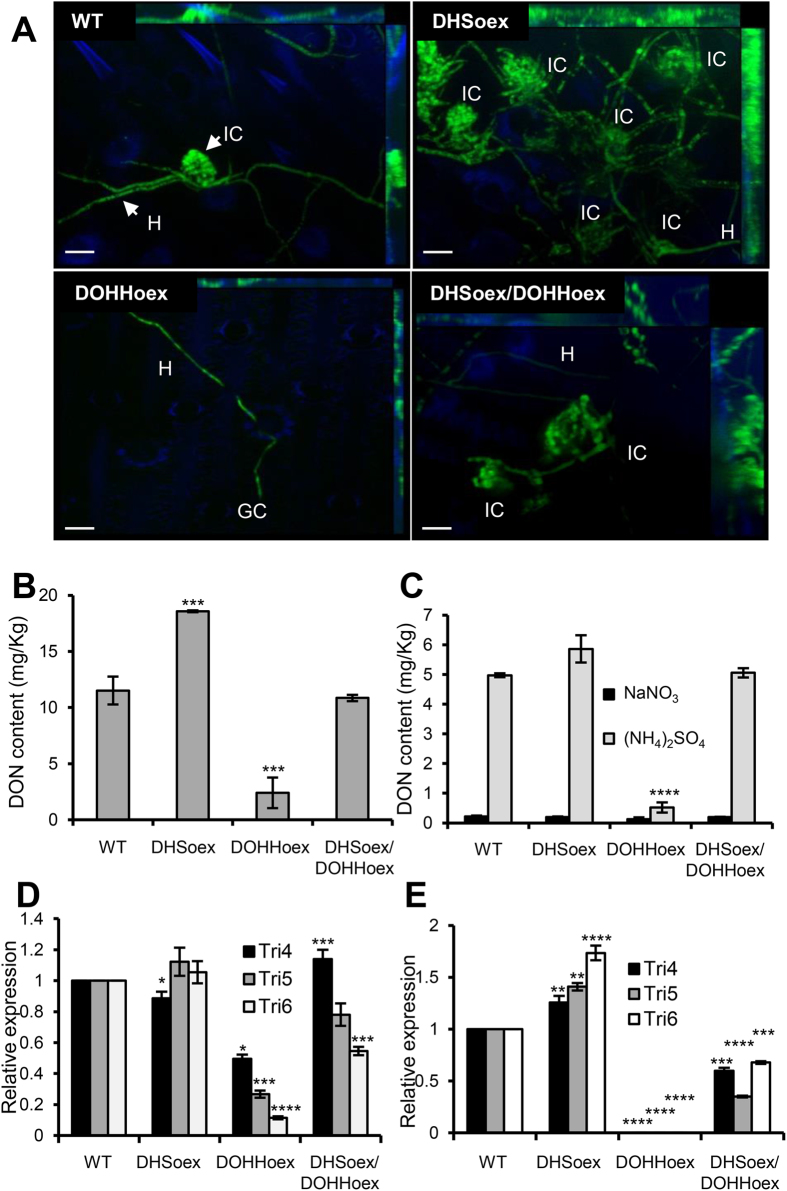
*DHS* and *DOHH* overexpression affects infection structure formation and impairs DON mycotoxin production. (**A**) Glumes inoculated with 5 μl of 2 × 10^4^ conidia per ml suspension of WT, DHSoex, DOHHoex or DHSoex/DOHHoex strains were incubated in a chamber with a photoperiod of 16 hours light at 16 °C. Complex infection structures were visualized 8 dpi. Maximum intensity projections of a z-stack of 20–30 pictures were produced with a Zeiss Axio Imager.Z1 microscope equipped with a Zeiss Apotome. The DHSoex mutant forms more complex infection structures than the wild type strain. In contrast, the DOHHoex mutant produced no infection structures. Hypha (H), complex infection structure (IC). Scale bar = 20 μm. Overexpression of *DHS* increases DON production as well as expression of the *TRI* genes in comparison to the wild type strain. On the contrary, *DOHH* overexpression suppresses production of DON mycotoxin and expression of the *TRI4*, *TRI5* and *TRI6* genes. DON mycotoxin production was measured in the overexpressing mutants grown in **(B)** wheat plants (cv. Nandu) for 21 days or **(C)** mycelia grown under inductive (5 mM (NH_4_)_2_SO_4_ as the nitrogen source) and non-inductive (5 mM NaNO_3_ as the nitrogen source) conditions at 28 °C, 150 rpm and 8 dpi. **(D)** RNA extracted from wheat plants infected with the wild type and overexpressing mutants 12 dpi was used to quantify the relative expression of *TRI4*, *TRI5* and *TRI6* genes. **(E)** Relative expression of TRI genes in culture was measured from mycelia grown under inductive conditions at 28 °C, 150 rpm and 3 dpi. Error bars indicate standard deviations calculated from data per triplicate samples and are representative of three independent experiments. Significance with respect to wild type: *p < 0.05, **p < 0.01, ***p < 0.001, ****p < 0.0001 (calculated with ANOVA-Bonferroni-Holm).

To investigate whether loss of virulence in DOHHoex or hypervirulence in DHSoex mutants is due to a lack or hyper-formation of infection structures as well as DON production, we screened infection during the second stage as described by Boenisch and Schäfer^21^. Detached wheat flower leaves were inoculated with the overexpressing mutants carrying a constitutive EGFP allele. Infection structure formation was determined at 8 dpi. Compared to the wild type, the DHSoex mutant developed 25% more complex infection structures. The DOHHoex mutant produced no infection structures, and only a few germinated conidia with epiphytical growth were detected. The double overexpressing mutant developed a similar number of infection structures as the wild type (Fig. 4A).

Production of DON mycotoxin in plants was measured on infected wheat spikes (cv. Nandu) at 21 dpi using a DON ELISA kit. Levels of DON production were normalized with the amount of fungal gDNA in the samples. During infection of wheat, the DHSoex mutant produced more DON (18 mg of DON/kg of *Fusarium*) than the wild type strain, whereas the DOHHoex mutant produced less DON under the same conditions (4 mg/kg). The double overexpressing mutant produced similar amounts of DON as the wild type strain (12 mg/kg) (Fig. 4B). In addition, levels of DON were determined in culture by inducing DON production in the wild type and overexpressing mutants grown for 8 days on minimal media containing 5 mM ammonium sulphate as the sole nitrogen source and 1% sucrose as a carbon source^19^. The DHSoex mutant produced slightly more DON in culture (6 mg/Kg) than the wild type (5 mg/kg). In contrast, the DOHHoex mutant showed no induction of DON, while the DHSoex/DOHHoex mutant produced levels of DON similar to the wild type (5.5 mg/kg). As a control, 5 mM sodium nitrate was used as a non-inductive nitrogen source (Fig. 4C).

Besides DON production, *TRI4*, *TRI5* and *TRI6* transcripts were quantified by real time quantitative polymerase chain reaction (RT-qPCR) during infection of wheat and in culture. RNA was extracted from wheat plants infected with the overexpressing mutants or the wild type strain, wheat inoculated with water was used as a negative control. β-tubulin (*TUB*) and ubiquitin-ligase (*UBI*) genes were used as normalizers in RT-qPCR. They were compared with 4 different methods to determine the best and more stable house-keeping gene for the expression assays. Both genes showed equal expression stability under the given conditions and *TUB* was used to normalize relative expression (Supplementary Tables S4 and S5). For in culture assays conidia were inoculated on minimal media under inductive conditions (as described above) and incubated for 3 days at 28 °C and 150 rpm. Expression levels of *TRI4, TRI5* and *TRI6* genes in the overexpressing mutants were compared to the transcript levels in the wild type, which were set to one. In plants, expression of the *TRI* genes was similar in the wild type, DHSoex and double overexpressing strains. However, with the DOHHoex mutant expression levels of the *TRI* genes were greatly reduced (Fig. 4D). In culture transcript levels of *TRI* genes were slightly higher in the DHSoex mutant and slightly lower in the DHSoex/DOHHoex mutant compared to the wild type, no *TRI5* or *TRI6* transcripts were detected in the DOHHoex mutant (Fig. 4E).

To determine whether the growth defect of the DOHHoex mutant on detached wheat flower leaves or during infection is due to a lack of secretion of degrading enzymes, the wild type and overexpressing mutants were grown on minimal media containing different carbon sources (e.g. wheat germ oil, Supplementary Table S2). No growth differences were observed, except when they were grown on 1% linoleic acid where interestingly, the DOHHoex mutant grew best (Supplementary Fig. S8).

### DOHHoex does not infect maize

**Figure 5:**
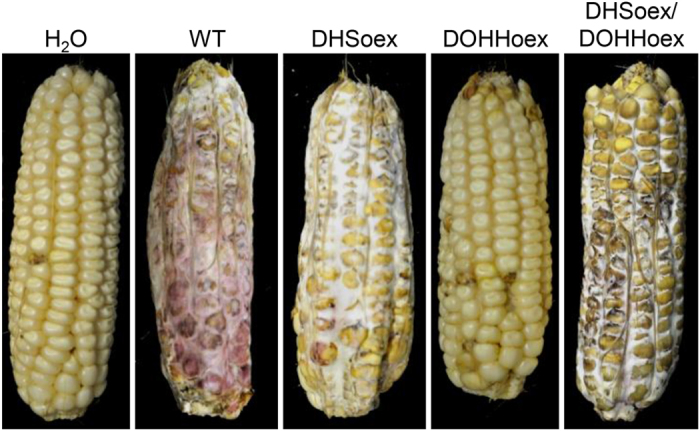
The DOHHoex overexpressing mutant has low virulence towards maize . Maize plants from the inbred line A188 were grown in a greenhouse until silks were visible. Silks were pollinated manually once per day for 3 days, then infected through the silk channel by injecting 4 ml of a suspension of 4 × 10^4^ conidia per ml. Water was used as a negative control. Symptoms were evaluated 35 dpi. While the DHSoex, DHSoex/DOHHoex and wild type strains fully infected maize cobs, the DOHHoex mutant shows few or no symptoms of *F. graminearum* infection. Images are representative of 8 repetitions per strain.

To establish whether the overexpressing mutants have similar effects on pathogenicity in maize, we carried out infection experiments with the inbred maize line A188^22^. Maize cobs were inoculated through the silk channel with the fungal strains, or water as a negative control. The severity of the symptoms were evaluated 35 dpi using the 7-class rating scale (disease severity rating = dsr) from Reid and Hamilton^23^, where 1 = no symptoms, 2 = 1–3%, 3 = 4–10%, 4 = 11–25%, 5 = 26–50%, 6 = 51–75% and 7 = more than 75% of kernels visibly mouldy. The water control showed no infection symptoms (dsr = 1). While the wild type, DHSoex and DHSoex/DOHHoex strains infected maize cobs completely (dsr = 7), the DOHHoex mutant produced few disease symptoms (dsr = 2) (Fig. 5).

### DOHHoex leads to complete hypusination of eIF5A

**Figure 6:**
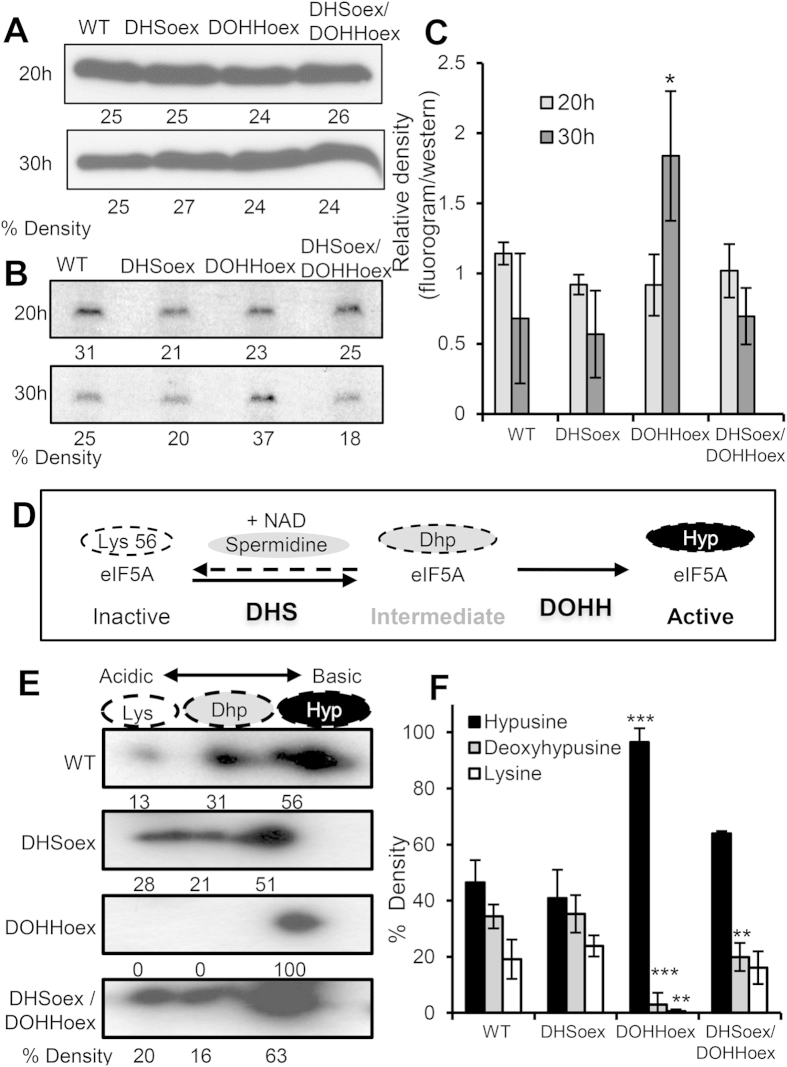
The DOHHoex mutant incorporates more hypusine into eIF5A. A crude extract of proteins was extracted from mycelia grown for 20 or 30 hours on CM supplemented with 70 μCi of [3H]-spermidine trihydrochloride. (**A**) Total amounts of eIF5A were determined by Western blotting with an anti-eIF5A antibody, which detected similar amounts of eIF5A protein produced in wild type and overexpressing mutants after 20 h or 30 h of growth in CM. (**B**) Wild type and overexpressing mutants incorporated similar levels of radiolabelled hypusine after 20 h of growth in CM. However, the DOHHoex mutant incorporated more spermidine after 30 h of growth. (**C**) The relative un-calibrated density was calculated using *ImageJ* software. The graph plots the percentage of fluorogram densities (hypusinated eIF5A) divided by the percentage of Western blot densities (total amount of eIF5A). Error bars indicate ± SD calculated from data of three independent experiments. (**D**) The eIF5A hypusination pathway depicting the DHS bi-directionality and DOHH uni-directionality (modified from 11). (**E**) The hypusination state of eIF5A in the overexpressing mutants was determined by 2D gel electrophoresis and subsequent Western blots using eIF5A antibody. The three isoforms of eIF5A, inactive (Lys), intermediate deoxyhypusine (Dhp) and active hypusine (Hyp) are detected in the wild type, DHSoex and DHSoex/DOHHoex strains, while the DOHHoex mutant contains only the fully hypusinated (active) form of eIF5A. (**F**) Percentage un-calibrated density was calculated using *ImageJ*. Error bars indicate ± SD calculated from data of three independent experiments (n = 3). Significance with respect to wild type: *p < 0.05, **p < 0.01, ***p < 0.001 (calculated with ANOVA-Bonferroni-Holm).

To investigate hypusine biogenesis in the wild type and overexpressing mutants, we determined spermidine incorporation by supplementing complete media with 70 μCi of [1,8–^3^H]-spermidine trihydrochloride. Similar amounts of total eIF5A protein were produced in the wild type and overexpressing mutants when grown for 20 or 30 hours in supplemented complete media (Fig. 6A). Incorporation of radiolabelled spermidine after 20 h of growth was also similar (Fig. 6B). However, after 30 hours of growth, the DOHHoex mutant incorporated more spermidine than the wild type or DHSoex and DHSoex/DOHHoex mutants. Relative density quantification confirmed these results, and revealed that at 30 hours the DOHHoex mutant had incorporated twice the amount of spermidine as wild type, DHSoex or DHSoex/DOHHoex strains (Fig. 6C), posing the question of whether DOHH overexpression leads to higher deoxyhypusination and/or hypusination of eIF5A.

The eIF5A protein exists in three different forms, unmodified (Lys), deoxyhypusinated (Dhp) or hypusinated (Hyp) (Fig. 6D)^24^,^25^, which can be distinguished by their isoelectric points. To determine the hypusination forms of eIF5A in the wild type and overexpressing strains, we performed 2D gel electrophoresis and subsequent Western blot using antibody against human eIF5A. The wild type, DHSoex and DHSoex/DOHHoex strains produced all three isoforms of eIF5A when grown on complete media for 72 hours, while only the fully hypusinated form of eIF5A was detected in the DOHHoex mutant (Fig. 6E). Determined by 2D gel electrophoresis, the isoelectric points of the unhypusinated, deoxyhypusinated and hypusinated forms were 4.65, 5.2 and 5.45, respectively. Data from three independent assays analyzed with the *ImageJ* program^26^ indicated that the majority of antibody attached to the more basic fully hypusinated form in all strains. Nevertheless, the other two more acidic deoxyhypusinated and unmodified forms of eIF5A were detected in the wild type, DHSoex and DHSoex/DOHHoex strains, whereas the DOHHoex mutant showed only the fully hypusinated form of eIF5A (Fig. 6F). Complete images of Westerns blots and fluorograms are given in Supplementary Fig. S9.

## Discussion

Here we demonstrate the repercussions of unbalanced expression of the eIF5A activating enzymes DHS and DOHH, and the tight regulation of eIF5A hypusine modification in the necrotrophic fungus *F. graminearum* during both vegetative and sexual reproduction, as well as during plant infection.

*EIF5A*, *DHS* and *DOHH* genes exist in all eukaryotes and their protein sequences are highly conserved among different species. Although two or more eIF5A isoforms have been identified in all eukaryotes, including some fungi^27^,^28^, in the plant pathogen ascomycetes we found only one protein containing the hypusine domain. The second protein with similarity to eIF5A does not contain a hypusine domain and has evolved a different function, namely the woronin body major protein Hex1, unique for filamentous fungi (Supplementary Fig. S1)^29^. This indicates that plant pathogenic ascomycetes possess a unique copy of the eIF5A activated by hypusination on a specific lysine (Lys 56 in *F. graminearum*), making the hypusine-eIF5A system an excellent target for proliferation control.

The *DHS* and *DOHH* genes exist as a single highly conserved copy in most eukaryotes, including ascomycetes^10^,^11^. In *S. cerevisiae* deoxyhypusine synthase Dys1 is essential for cell survival; on the contrary deoxyhypusine hydroxylase Lia1 as well as its orthologue Mmd1 from *Schizosaccharomyces pombe* are not essential^30^,^31^. Unlike the yeast, *DHS* and *DOHH* gene deletions are lethal, suggesting full hypusination of eIF5A in *F. graminearum* is essential for cell viability. Surprisingly, overexpressing *DHS* or *DOHH* individually resulted in opposing phenotypes. In general, *DHS* overexpression led to an increase in virulence traits, whereas overexpression of *DOHH* reduced virulence. The simultaneous expression of both enzymes restored wild type phenotypes.

Expression of *EIF5A* gene was unaffected in the overexpressing mutants. Similarly the expression of the *DHS* gene was unchanged in the DOHHoex mutant and expression of *DOHH* gene was unchanged in the DHSoex mutant. These results indicated that no transcriptional co-regulation occurs among the genes necessary for hypusine biosynthesis and their substrate eIF5A. The regulatory effect of DHS and DOHH towards eIF5A is only at a translational level under the studied conditions.

Conidia are the infectious agents of fungi, essential for survival, proliferation and virulence^32^. The observed increased or impaired production of asexual conidia as a result of overexpressing *DHS* or *DOHH* would lead to a significant advantage or disadvantage in the field. Besides propagation, fungal pathogens must overcome hostile environments that include stress insults deriving from the host during invasion^33^,^34^, as well as toxic external compounds or by products of its own metabolism, such as ROS generated during aerobic respiration or infection^35^,^36^. Interestingly, the DOHHoex mutant was more sensitive to hyperosmotic and cell wall stresses, and notably produced extremely high levels of ROS.

The DOHHoex mutant exhibited both decreased production of asexual conidia and increased production of perithecia. Although the harmful effect of ROS due to uncontrolled oxidizing reactions affecting DNA, RNA, proteins and lipids have been documented, ROS has been found to be essential for sexual development^35^,^36^. In *Botritis cinearea* as well as in *F. graminearum*, deletion of the NADPH oxidase NoxA results in low ROS and no perithecia production, emphasizing the essential role of ROS during sexual reproduction^37^,^38^.

The pleiotropic stress responses could be due to direct involvement of eIF5A in diverse signalling pathways. In yeast, overexpression of protein kinase C (PKC1), PKC1 regulators WSC1, WSC2 and WSC3, and diverse regulators of actin organization restored the growth of the thermo-sensitive mutant *tif51A* (orthologue of *F. graminearum EIF5A*), suggesting a role for eIF5A in cell integrity and establishing actin polarity^4^,^5^. Moreover, lack of equilibrium in ROS production is one of the main consequences of deleting the gene *FgOS2*, which encodes a MAP kinase necessary for stress responses, or the downstream transcription factors *FgATF1* and *FgAP1* in *F. graminearum*^39^,^40^,^41^. Therefore, eIF5A hypusination is likely to directly or indirectly influence signalling pathways.

Imbalances in hypusination of eIF5A have not been investigated until now, nor the role of eIF5A, DHS and DOHH in fungal pathogenicity. Fusarium Head Blight disease is initiated by airborne spores landing on open florets during anthesis^13^,^14^. Open florets allow *F. graminearum* to make contact with young anthers, developing caryopses and adaxial surfaces of lemma, palea and glumes. Subsequent access is provided through vascular tissue crossing the rachis node to the rachis. From the rachis, *F. graminearum* then extends to other florets, aggravating the disease^16^,^19^. Point inoculation of wheat spikelets during anthesis and silk inoculation of maize with the single overexpressing mutants resulted in strikingly opposite phenotypes. *DHS* overexpression led to hyper-virulence in wheat and wild type infection rates in maize. On the contrary, *DOHH* overexpression abolished infection symptoms on wheat and maize. The DHSoex/DOHHoex mutant induced symptoms similar to wild type on both wheat and maize.

Analysis of infection progression on wheat using constitutive EGFP-tagged strains demonstrated that *DHS* overexpression drives faster progression than the wild type, while *DOHH* overexpression led to weak colonization of the inoculated spikelet, resembling typical type II resistance, with progression stopping at the base of the rachis node.

Production of the trichothecene mycotoxin DON is necessary for full *F. graminearum* invasion of wheat but not maize^15^. The rachis node was reported to induce expression of the *TRI5* gene, which encodes a trichodiene synthase, a key enzyme in the trichothecene pathway^19^. Moreover, a strain lacking the *TRI5* gene is prevented from moving into the rachis by thickening of the cell walls, demonstrating that the rachis node is a key component of wheat resistance against *F. graminearum*^16^. Our virulence results could at least partially be explained by the elevated production of DON associated with *DHS* overexpression and the low levels of DON production with *DOHH* overexpression during plant infection. The differences in DON regulation occur at the transcriptional level, with an up-regulation of genes in the DHSoex mutant and a dramatic down-regulation in the DOHHoex mutant.

Although DON production partly explains the hyper and hypo-virulence phenotypes of DHSoex and DOHHoex mutants in wheat, DON mycotoxin is not essential for maize infection. Therefore, the avirulence of the DOHHoex mutant in maize may be due to the increased ROS production in this mutant. As mentioned above, deletion of the stress activated protein kinase FgOS-2 in *F. graminearum* led to a massive release of ROS after mild osmotic stress, drastically reduced DON production, and avirulence on maize^39^, closely resembling the phenotypes of *DOHH* overexpression. This indicates the potential involvement of eIF5A in signalling pathways and the importance of ROS production.

*F. graminearum* initially colonizes the surface of wheat florets where conidia germinate epiphytically without immediate penetration. After colonization of the leaf surface, fungal hyphae undergo morphological differentiation and form infection structures, whose complexity increases with time^21^. Indeed, the hypervirulent DHSoex mutant exhibited profuse mycelia growth and infection structure formation. *DOHH* overexpression was associated with less dense mycelia growth and no infection structures forming on the flower leaf surface. Interestingly, the development of infection structures as well as the pathogenesis of *F. graminearum* is regulated by the cAMP cyclic response pathway^42^ and the mitogen-activated protein kinase (MAPK) gpmk1^43^. Again, these results are in agreement with our hypothesis of eIF5A being involved in diverse signalling pathways.

In the rice blast fungus *M. oryzae*, a Δ*cpkA* mutant lacking protein kinase A and a Δ*gpmk1* mutant lacking the MAP kinase involved in the cascade that responds to hard, hydrophobic surfaces, or the absence of exogenous nutrients, are unable to develop appressoria, and consequently, to invade rice leaves^44^. In the southern corn blight pathogen *Cochliobolus heterostrophus* pmk1 is necessary for appressoria development^45^; in *F. oxysporum* the pmk1 orthologue fmk1 is required for pathogenicity^46^. In *F. graminearum* the Δ*gpmk1* mutant is defective in degrading host cell wall material, and fails to induce secreted lipolytic activity^47^.

An additional reason for the differences in infection structure formation and pathogenicity could be that cell cycle progression from the G1/S phase is impaired in the DOHH overexpressing mutants^1^,^2^. In *M. oryzae* G1/S cell cycle progression has been implicated in appressoria formation. Conditional *nim1*^*I*327E^ mutants that arrest entry into S-phase failed to differentiate into functional infection cells, i.e. appressoria on hydrophobic surfaces. Instead, germlings continue growing with a filamentous morphology^48^. As mentioned before, eIF5A has been implicated in cell-cycle transition from G1 to S-phase by allowing selective translation of mRNAs encoding proteins specialized in initiating and enabling eukaryotic proliferation^1^,^2^. Results from overexpressing *DHS* or *DOHH* suggest that the imbalance of these activating enzymes causes differences in hypusine formation that impair or activate the mRNA transport and translation necessary for the morphological transition during early infection of wheat.

We tested how the expression rate of the different hypusine-forming enzymes influences final hypusine biogenesis in eIF5A by incorporating [1,8-^3^H]-spermidine. The DOHHoex mutant incorporated 2-fold more spermidine after 30 hours of growth, which turned out to be due to full hypusine formation. All forms, unmodified, deoxyhypusinated, or hypusinated eIF5A proteins were found in the wild type, DHSoex and DHSoex/DOHHoex strains, with the fully hypusinated protein being the major form. However, DOHHoex mutant extracts only contained the fully hypusinated-eIF5A protein. This could be due to the non-reversible DOHH reaction with its substrate Dhp-eIF5A, exacerbated by amplification of the second enzymatic step, locking eIF5A into its hypusinated form^11^. In contrast, the DHS reaction is reversible and amplification of the first enzymatic step does not lock eIF5A in its deoxyhypusinated form (Fig. 6D)^49^.

The observed phenotypes may be due to the highly hypusinated eIF5A, and/or to the absence of the unhypusinated and deoxyhypusinated forms of eIF5A (Table 1). The latter would point to additional biological functions of these proteins. We and others used inhibitors of DHS to abolish hypusination of eIF5A and inhibit a pathogenic phenotype. However, cells very often overcome inhibitors by overexpressing the genes encoding the inhibited enzyme^50^. Here we demonstrated that in such a case *DHS* overexpression will result in unwanted hypervirulence. In contrast, *DOHH* overexpression leads to hypovirulence of *F. graminearum*. Intriguingly, inhibitors directed at DOHH may offer a way to imbalance eIF5A hypusination; selection for resistance, and hence overexpression would lead to the desired phenotype even upon removing the inhibitor.

## Methods

A full description of material and methods is provided in Supplementary Material and Methods.

### *DHS, DOHH* cloning and overexpression

1077 bp or 1014 bp cDNA fragments containing the ORFs of the submitted *DHS* or *DOHH* genes from the *F. graminearum* wild type strain 8/1^51^ were amplified with a primer pair incorporating restriction sites (*BamH* I for *DHS* and *Sac* I and *Xba* I for *DOHH*; Supplementary Table S3). The PCR products were cloned into the pJET1.2 vector (Fermentas-Thermo Scientific, Germany) according to the manufacturer’s instructions and sequenced. For overexpression of *DHS*, pMW-DHS (9277 bp) was constructed by inserting the PCR product into the *BamH* I site of the pCWHyg-PgpdA vector, between the gpdA promoter and trpC terminator from *Aspergillus nidulans*, using standard protocols^52^. For overexpression of the *DOHH* gene, encoding a HEAT-repeat protein^53^, the plasmid pMW-DOHH (6971 bp) was constructed by inserting the ORF of *DOHH* into the *Sac*I and *Xba*I restriction sites located between the gpd1 promoter and trpC terminator from *Cochliobolus hetrostophus* of the pII99-Pgpd1 vector containing the geneticin resistance cassette (Supplementary Table S1). *F. graminearum* was transformed with the pMW-DHS vector linearized with *Cla*I, which cuts the *DHS* sequence once at position 328, or the pMW-DOHH plasmid linearized with *Bbs* I, which cuts the *DOHH* sequence once at position 556, to induce a single crossover event. Gel electrophoresis, restriction enzyme digestion, Southern blots and sequencing were performed using standard procedures. The double overexpressing mutant was generated by introducing the pMW-DOHH plasmid linearized with *Bbs* I into the DHSoex mutant (Supplementary Table S1 and Fig. S3).

### Wheat spike and detached wheat flower leaves infection assays

Spike infection and detached wheat flower leaves infection assays were prepared according to Boenisch and Schäfer^21^. Briefly, for intact plant infection, wheat spikes of the susceptible cultivar Nandu and partially resistant cultivar Melissos were inoculated at two of the middle florets between lemma and palea with 10 μl of a 2 × 10^4^ conidia per ml suspension; infection was analyzed 7, 14 and 21 days post inoculation (dpi) or at indicated time points. The degree of infection was determined as follows: % Infection = (number of infected spikelets ∗ 100)/ total number of spikelets. Ten spikes were used for each assay. Each assay was repeated at least three times. For the detached wheat flower leaves assay, single glumes from the susceptible cultivar Nandu were isolated and placed on 2% water-agar plates and inoculated with 5 μl of a 2 × 10^4^ conidia per ml suspension and incubated in a growth chamber with a 16 h light photoperiod at 16 °C–18 °C. Infection symptoms were analyzed 8 dpi. Sterile water was used as a negative control. Four Petri dishes contained 8 biological replicates of one floret organ and represented one independent experiment.

### Maize infection assay

To ensure good pollination, silks of maize cobs from the inbred line A188 were pollinated manually once per day for 3 days. Pollinated cobs were infected through the silk channel by injecting 4 ml of a 2 × 10^4^ conidia per ml suspension. 4 ml of sterile water were used as a negative control. Disease symptoms were analyzed 35 dpi^23^.

### Microscopy of infected wheat spikes and detached wheat flower leaves

Wheat heads were examined using a Zeiss MZFLIII microscope (Leica Microsystems, Switzerland) 3, 7 and 10 dpi. Fungal growth and plant necrosis were detected using bright field microscopy, and GFP was detected with a GFP2 filter, which allows visualization of chlorophyll auto-fluorescence in red and mycelia growth in green using the same filter. Photographs were taken with a Leica DFC500 digital colour camera. Leica LAS software (version 2.7.1) was used for image acquisition and processing. Microscopy of detached wheat flower leaves was carried out as described by Boenisch and Schäfer^21^. Briefly, infection structures of reporter strains were investigated by fluorescence microscopy using a Zeiss Axio Imager.Z1 microscope equipped with a Zeiss Apotome. An ultra violet lamp HAL 100 served as the UV light source. GFP was detected with an excitation 450–490 nm/emission 500–509 nm wavelength. The blue autofluorescence of the plant was detected in the 420 to 470 nm range. Images were taken with Zeiss AxioCam MRm CCD camera. Image processing and generation of maximum intensity projections (MIP) of z-stacks were performed with Zeiss AxioVision software (version 4.8.1).

### Measuring deoxynivalenol (DON) production

For in culture mycotoxin deoxynivalenol (DON) measurements, the wild type and overexpressing mutant strains were grown under inducing conditions: minimal media with 1% sucrose as a sole carbon source and 5 mM ammonium sulphate as the nitrogen source, or non-inducing conditions: minimal media with 1% sucrose and 5 mM sodium nitrate, over 3 days at 28 °C and 180 rpm on a rotary shaker. 20 mg of dried mycelia were used for measuring DON production. For DON measurements during infection, wheat spikes inoculated as described above were harvested 21 days after inoculation and the 4 inoculated florets from the centre were used to evaluate DON by ELISA using the RIDASCREEN^®^ DON kit or the RIDASCREEN^®^ DON-FAST kit (R-Biopharm, Germany).

### NBT-staining and H_2_O_2_ production assays

For superoxide detection, 10^3^ conidia of the wild type or mutant strains were placed on CM agar plates and allowed to grow for 3 days at 28 °C in the dark. Mycelia were stained with a solution of nitroblue tetrazolium (0.2% NBT) in water, incubated at RT in the dark for 40 min and the reaction stopped with absolute ethanol. H_2_O_2_ was measured using 3-day-old mycelia grown on liquid CM. The mycelia were washed twice with sterile distilled water and harvested. 20 mg of mycelia were placed on a 96-well microtiter plate containing 200 μl of Krebs–Ringer phosphate buffer (KRPG: 145 mM NaCl, 5.7 mM sodium phosphate, 4.86 mM KCl, 0.54 mM CaCl2, 1.22 mM MgSO4, 5.5 mM glucose, pH 7). Amplex^®^ Red Hydrogen Peroxide/Peroxidase Assay Kit (Invitrogen, Darmstadt, Germany) was used to measure the H_2_O_2_ production following the manufacturer’s instructions. Measurements were performed 0.5, 1 or 1.5 h after the reaction started.

### eIF5A detection assay

For analysis of eIF5A protein levels, macroconidia were inoculated in 20 ml complete media at a final concentration of 5 × 10^5^ per ml and germinated for 20 h or 30 h at 28 °C and 150 rpm. Mycelia were filtered, frozen in liquid nitrogen, ground in a mortar and resuspended in a protein extraction buffer containing 10% sucrose, 50 mM Tris-HCl, pH 7.5, and a cocktail of proteases inhibitors for fungi extracts (Sigma-Aldrich, Germany). Samples were homogenized and incubated for 10 min on ice, centrifuged for 5 min at 4 °C to pellet cell debris and the protein concentration of the supernatant was determined using Bradford reagent (Sigma-Aldrich, Germany). 30 μg of total protein were separated on a 15% SDS-polyacrylamide gel and blotted onto a PVDF-Immobilon^®^ membrane (Millipore, Darmstadt, Germany) using standard protocols^52^. Membranes were blocked with 5% non-fat skimed milk (Fluka) in TBS-T for 1 h. eIF5A protein was detected using the eIF5A1 antibody (GTX111013-rabbit anti-human eIF5A1, Acris-antibodies GmbH (Herford, Germany) and a secondary anti-rabbit IgG, HRP-linked antibody (New England Biolabs GmbH, Frankfurt am Main, Germany). Detection was performed using Lumiglo reagent (New England Biolabs GmbH, Frankfurt am Main, Germany) following the manufacturer’s instructions.

### Detection of eIF5A precursors and the hypusine-eIF5A protein

Cell lysate (250 μg of protein) for two-dimensional gel electrophoresis were prepared as described above. The first dimension isoelectro-focusing was performed in the PROTEAN^®^ IEF Cell from BioRad, using 7 cm (pH range 5.5–6.8) ReadyStrip TM IPG Strip gel (Bio-Rad Laboratories, Munich, Germany). The proteins in the first dimension Strip gel were further separated in the second dimension by SDS-PAGE on a 15% polyacrylamide gel, and transferred on to a PVDF-Immobilon^®^ membrane (Millipore, Darmstadt, Germany) to detect the various forms of eIF5A. Western blotting was performed as described above.

### Hypusine formation assay

A conidial suspension of 5 × 10^5^ conidia was incubated in 20 ml complete media containing 70 μCi of [^3^H]-Spermidine Trihydrochloride (Perkin Elmer, Hamburg, Germany) with 150 rpm shaking at 28 °C. After 24 h (or indicated time points) of incubation the mycelia were harvested using Whatman filter paper. To produce protein crude extracts the samples were resuspended in protein extraction buffer (given above) followed by sonication on ice for 15 seconds using a 5000 micro tip (Branson Sonifier B12). 50 μg of protein extract were separated by 14% SDS-PAGE. The proteins were fixed by soaking the gel in 25% isopropanol/10% acetic acid (30 min), washed twice with distilled water and then soaked in 25 ml of amplify solution (GE Healthcare, Freiburg, Germany) for 30 min. The dried gel was exposed to an ultrasensitive X-ray film for 1 to 6 weeks. The percentage density of the radio labelled and Western blot signals were calculated with the *ImageJ* program^26^, and the relative density was determined by dividing the percentage density of eIF5A signals detected on the Western blot by the percentage density of incorporated spermidine detected on the autoradiogram.

## Additional Information

**Accession numbers**: The complete sequences of the EIF5A and DOHH cDNAs, as well as predicted proteins were submitted to Genbank (accession numbers: GU809213.1 for the EIF5A gene, GU735676.1 for the DOHH gene, ADE59476.1 for eIF5A protein and ADE61839.1 for DOHH protein). Gene numbers from Fusarium Comparative Database (BROAD INSTITUTE): β-tubulin (TUB) FGSG_06611, ubiquitin ligase (UBI) FGSG_08811, eukaryotic translation initiation factor 5A (EIF5A) FGSG_01955, deoxyhypusine synthase (DHS) FGSG_00323 and deoxyhypusine hydroxylase (DOHH) FGSG_09773.

**How to cite this article**: Martinez-Rocha, A. L. *et al*. Posttranslational hypusination of the eukaryotic translation initiation factor-5A regulates *Fusarium graminearum* virulence. *Sci. Rep*. **6**, 24698; doi: 10.1038/srep24698 (2016).

## Supplementary Material

Supplementary Information

## Figures and Tables

**Table 1:** Summary of DHSoex and DOHHoex hypusinated forms and phenotypes.

Genetic background	DHSoex	DOHHoex
Hypusination	WT-like	–
	Unhypusinated	N.d.
	Deoxyhypusinated	N. d.
	Hypusinated	Hypusinated
Phenotype	Hyper-virulence	Hypo-virulence
	More conidia^1^	Less conidia^1^
	WT-like ROS	More ROS
	More DON	Less DON
	More infection structures	No infection structures
	WT-like perithecia^2^	More perithecia^2^
N. d. = Not detected	^1^Asexual propagation	^2^Sexual propagation
