# Supplementary material for: Posttranslational hypusination of the eukaryotic translation initiation factor-5A regulates Fusarium graminearum virulence
Source: Sci Rep. 2016 Apr 21;6:24698. doi: 10.1038/srep24698 (PMC4838825; doi:10.1038/srep24698)
Supplement: Supplementary Information [file srep24698-s1.pdf]

Posttranslational hypusination of the eukaryotic translation initiation factor-5A regulates *Fusarium graminearum* virulence

Authors:

Ana Lilia Martinez-Rocha[1,+,*], Mayada Woriedh[1,4,+], Jan Chemnitz[2], Peter Willingmann[1], Cathrin Kröger[1], Birgit Hadeler[1], Joachim Hauber [2,3], Wilhelm Schäfer[1]

Affiliations:

[1]University of Hamburg, Biocenter Klein Flottbek, Molecular Phytopathology and Genetics, Hamburg, D-22609, Germany.

[2]Heinrich Pette Institute, Leibniz Institute for Experimental Virology, Department Antiviral Strategies, Hamburg, D-20251, Germany.

[3]German Center for Infection Research (DZIF), partner site Hamburg, Hamburg, Germany.

[4]Current address: University of Regensburg, Cell Biology and Plant Biochemistry, Regensburg, D-93053, Germany.

* **Corresponding author:**

ALMR, E-mail: ana.lilia.martinez-rocha@uni-hamburg.de , Tel: (+49) 40 42 816 309

Supplementary Information

Supplementary Fig. S1 Alignment of amino acid sequences of eIF5A and DOHH.

Supplementary Fig. S2 Targeted gene deletion of *DOHH* is not viable in *F. graminearum*.

Supplementary Fig. S3 Cloning and overexpression of *DHS* and *DOHH*.

Supplementary Fig. S4 Protein levels of DHS or DOHH in the wild type and overexpressing mutants.

Supplementary Fig. S5 *DHS* and *DOHH* overexpression impair asexual and sexual conidia production and germination.

Supplementary Fig. S6 Overexpressing mutants responsible for eIF5A hypusination are impaired in stress responses.

Supplementary Fig. S7 Construction and introduction of eGFP into the overexpressing mutants.

Supplementary Fig. S8 Linoleic acid promotes growth on the DOHHoex mutant.

Supplementary Fig. S9 Complete Western blots and fluorograms from Figure 6.

Supplementary Table. S1 Plasmids used or generated in this work.

Supplementary Table. S2 Growth of wild type and overexpressing mutants under different media conditions.

Supplementary Table. S3 Oligonucleotides used in this work.

Supplementary Table. S4 Comparison of gene stability (M value) of two housekeeping genes for internal control.

Supplementary Table. S5 Statistics of β-Tub and Ubi genes for internal control based on results provided by software BestKeeper.

Supplementary Material and Methods

Supplementary Fig. S1

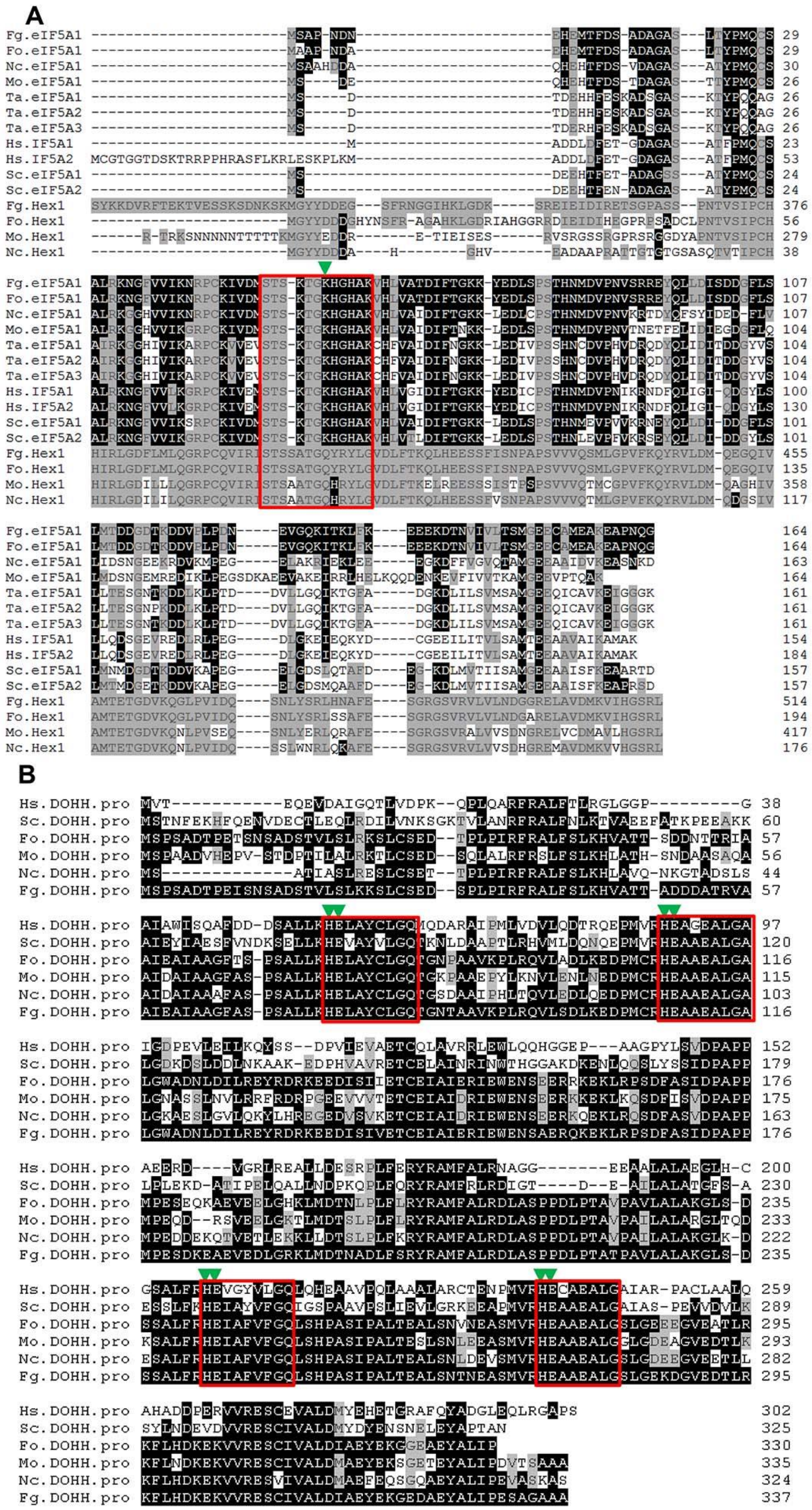

Supplementary Fig. S1 Alignment of amino acid sequences of eIF5A and DOHH. The eIF5A or DOHH amino acid (aa) sequences were aligned with clustal W. **(A)** The putative aa sequence of the eukaryotic translation initiation factor 5A (eIF5A1, GenBank: ADE59476.1) and the second protein with similarity to eIF5A, Hex1 (Hex1, GenBank: ESU14566.1) from *F. graminearum* (Fg) were aligned with protein sequences of eIF5A1 (NCBI Ref Seq: NP_001093162.1) and eIF5A2 isoforms (NCBI Ref Seq: NP_065123.1) from *Homo sapiens* (Hp); eIF5A1 (Hyp2, GenBank: AAB65008.1) and eIF5A2 (GenBank: AAA35156.1) from *Saccharomyces cerevisiae* (Sc); eIF5A1 (GenBank: AAA61707.1) and Hex1 (GenBank: EAA34471.1) from *Neurospora crassa* (Nc); eIF5A1 (GenBank: EHA52059.1) and Hex1 (GenBank: EHA46326.1) from *Magnaporthe oryzae* (Mo); eIF5A1 (GenBank: EGU78279.1) and Hex1 (GenBank: EGU80510.1) from *Fusarium oxysporum* (Fo) and finally eIF5A1 (GenBank: AAZ95171.1), eIF5A2 (GenBank: AAZ95172.1) and eIF5A3 (GenBank: AAZ95173.1) from *Triticum aestivum* (Ta). The hypusine modification site (red box) is located at the basic N-terminal domain exposed loop (hypusine loop 50-61 aa in *F. graminearum* eIF5A1). A green arrow-head indicates the specific lysine where hypusination occurs (Lys 56 in *F. graminearum*). Amino acid residues identical to *F. graminearum* eIF5A1 are in black. Amino acid residues identical to *F. graminearum* Hex1 are in grey. Identity percentages between eIF5A proteins and eIF5A1 from *F. graminearum* range from 54.1 % with *T. aestivum* eIF5A-2 to 98% with *F. oxysporum* eIF5A1. Identity percentage between eIF5A-1 and Hex1 proteins from *F. graminearum* is 23.9%. **(B)** The putative aa sequence of the deoxyhypusine hydroxylase (DOHH, GenBank: ADE61839.1) from *F. graminearum* was aligned with the predicted DOHH proteins from *H. sapiens* (NCBI Reference Sequence: NP_112594.1); DOHH (Lia1p) from *S. cerevisiae* (GenBank: DAA08857.1); DOHH from *N. crassa* (NCU05252.7), DOHH from *M. oryzae* (MGG_01725.7), and finally DOHH from *F. oxysporum* (FOXG_08287.2). DOHH is a HEAT-repeat protein containing four His-Glu motifs (red boxes). It contains eight conserved critical amino acids HE (green arrow-heads) and two potential iron coordination sites (53). Identity percentages between the DOHH proteins and *F. graminearum* DOHH range from 45.5% with *H. sapiens* DOHH to 91.5% with *F. oxysporum* DOHH.

Supplementary Fig. S2

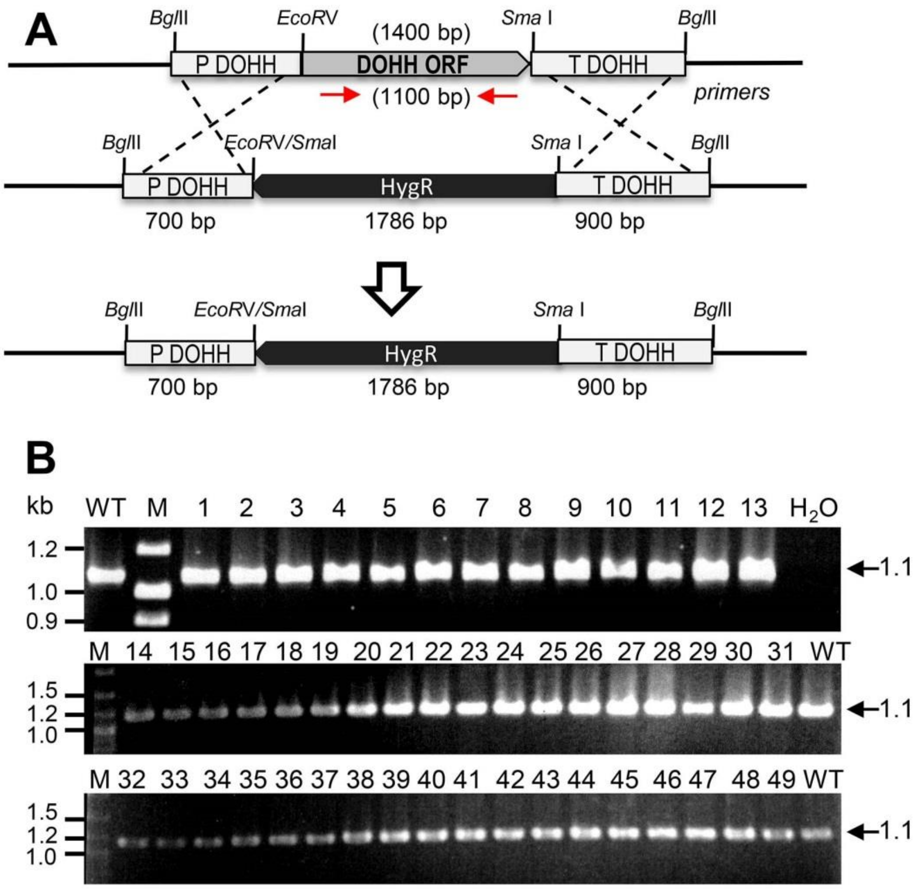

Supplementary Fig. S2 Targeted gene deletion of *DOHH* is not viable in *F. graminearum*. (A) Schematic representation of targeted gene replacement strategy. A fragment containing the *DOHH*-ORF, the 5` and 3` flanks was amplified from genomic DNA with specific primers (Supplementary Table S3) and cloned into pJET1.2 blunt cloning vector (Fermentas-ThermoScientific, Germany). A 1.4 kb fragment of the *DOHH* ORF was removed by using the enzymes *EcoR* V and *Sma* I which produce blunt ends. A 1.78 kb hygromycin B resistance cassette with incorporated *Sma*I sites replaced the *DOHH* 1.4 kb fragment to create the deletion vector pMW-DOHH-Hyg (Supplementary Table S1). The final linear deletion cassette was released with *Bgl* II restriction sites and used to transform *F. graminearum* wild type (8/1). **(B)** Amplification of DOHH fragments by PCR with specific primers from the *DOHH* coding region (red arrows) and genomic DNA from at least 49 transformants and the wild type strain resulted in amplification of the original 1.1 kb fragment of the *DOHH* ORF, indicating that non- homologous recombination had taken place. M: DNA ladder, WT: wild type, 1-49: transformants with non-homologous recombination, H_2O: water used as a template for negative control.

Supplementary Fig. S3

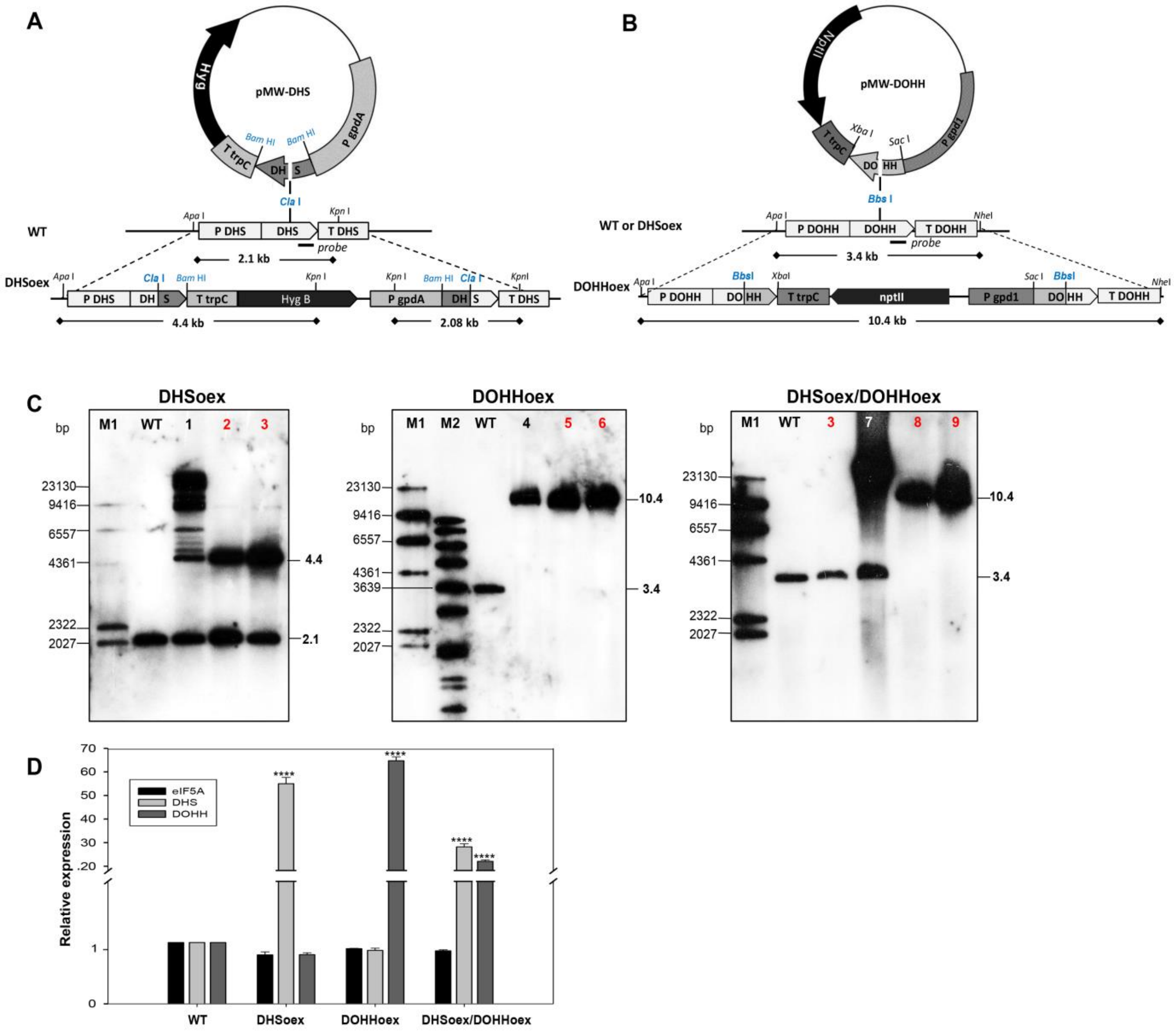

Supplementary Fig. S3 Cloning and overexpression of *DHS* and *DOHH*. (A, B) Schematic representation of cloning strategies and gene integration events. **(A)** The *DHS* ORF was amplified from cDNA with specific primers and inserted with *BamH* I sites into the pCWHyg-PgpdA vector in frame with the PgpdA promoter to generate the pMW-DHS vector. The pMW-DHS vector was linearized with restriction enzyme *Cla* I in the *DHS* ORF and used to transform *F. graminearum* wild type, resulting in a single crossover event with targeted genomic sequences. **(B)** The *DOHH* ORF was amplified from cDNA with specific primers and inserted with *Sac* I and *Xba* I sites into the pII99-Pgpd1 vector in frame with the Pgpd1 promoter to generate the pMW-DOHH vector. The pMW-DOHH vector was linearized with *Bbs* I to transform *F. graminearum* wild type, resulting in a single crossover event with targeted genomic sequences. Single crossover leads to a duplication of the ORF, one copy under the native promoter (PDHS or PDOHH) and the second under the constitutive gpd (PgpdA or Pgpd1) promoter. **(C)** Southern blots confirm homologous recombination with a single crossover of PgpdA:DHS or Pgpd1:DOHH. The restriction enzymes *Apa* I and *Kpn* I were used to digest gDNA

from the wild type and DHSoex mutants. *Apa* I and *Nhe* I restriction enzymes were used to digest gDNA from the wild type and DOHHoex mutants. A *DHS* fragment was used as a probe for DHSoex mutants and a *DOHH* fragment for DOHHoex and double overexpressing mutants. The double overexpressing DHSoex/DOHHoex mutants were constructed by introducing the pMW-DOHH linearized vector into the DHSoex mutant shown in lane 3. Lanes, M1: DNA molecular weight marker II, DIG-labeled; WT: wild type; M2: DNA molecular weight marker VII, DIG-labeled; 1-3: DHSoex mutants, 4-6: DOHHoex mutants; 7-9: *DHSoex/DOHHoex* mutants. Selected transformants for further experiments are shown in red. **(D)** *DHS* and *DOHH* gene expression was determined by quantitative RT-PCR. RNA was extracted from the wild type strain, DHSoex, DOHHoex and DHSoex/DOHHoex mutants grown on CM for 24 h. Significance with respect to wild type: **** $p<0.0001$ (calculated with ANOVA-Bonferroni-Holm).

Supplementary Fig. S4

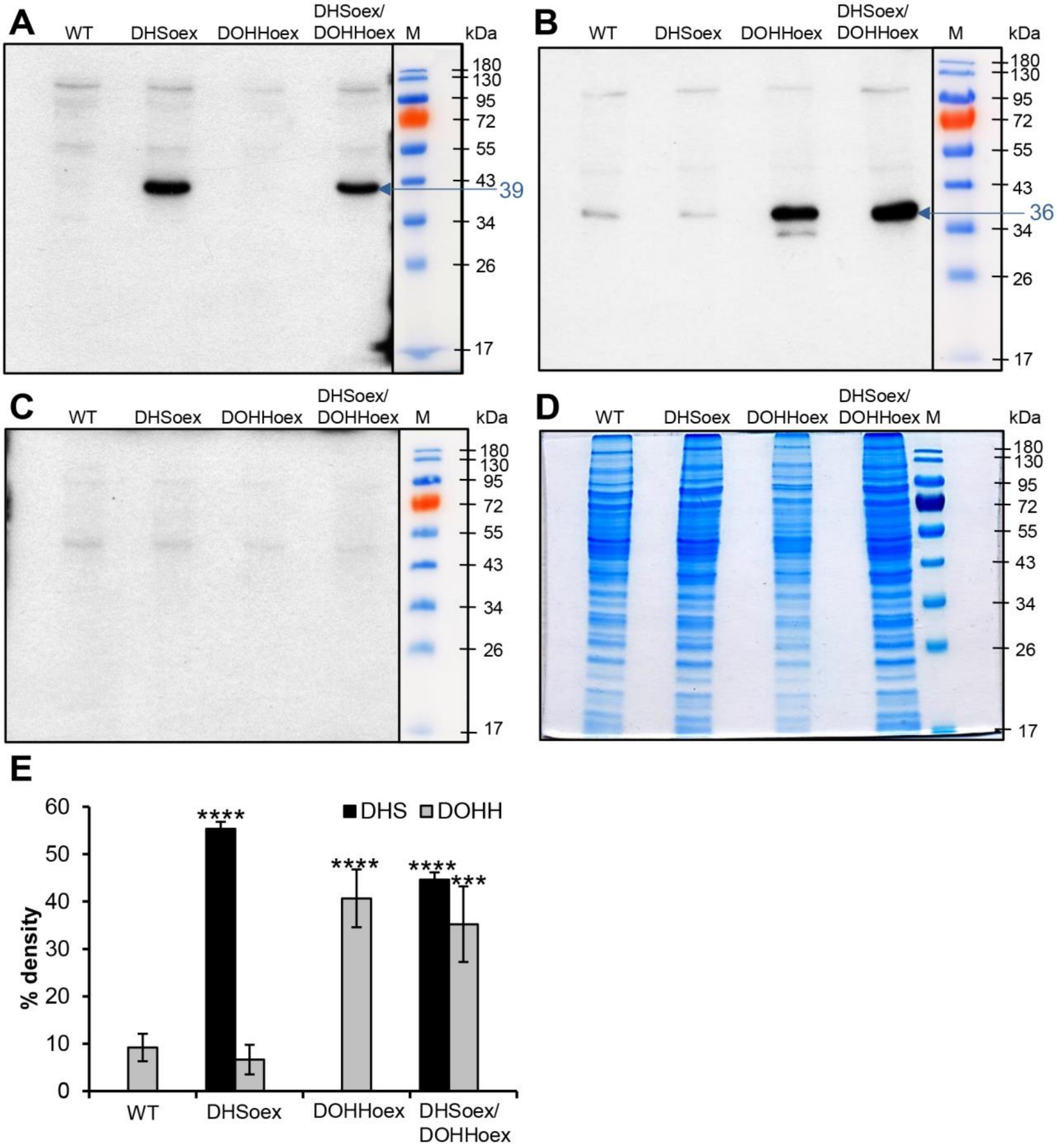

Supplementary Fig. S4 Protein levels of DHS or DOHH in the wild type strain and overexpressing mutants. High levels of DHS or DOHH proteins are present in the respective overexpressing mutants compared to the wild type strain. The predicted size for *F. graminearum* DHS protein is 39 kDa and 36kDa for DOHH protein. 10µg of proteins extracted from mycelia grown on complete media during 24h, at 28°C and 150 rpm were used to prepare western blots with **(A)** anti-human DHS (1:1000), **(B)** anti *F. graminearum* DOHH (1:2000), or **(C)** pre-immune serum (1:2000). **(D)** Coomassie staining (SDS-PAGE 12.5%) shows similar amount of loaded protein. **(E)** The intensity of the bands was quantified with the program *imageJ*. Standard deviation comes from two biological replicates and two technical replicates. Significance with respect to wild type: *** $p<0.001$, **** $p<0.0001$ (calculated with ANOVA-Bonferroni-Holm).

Supplementary Fig. S5

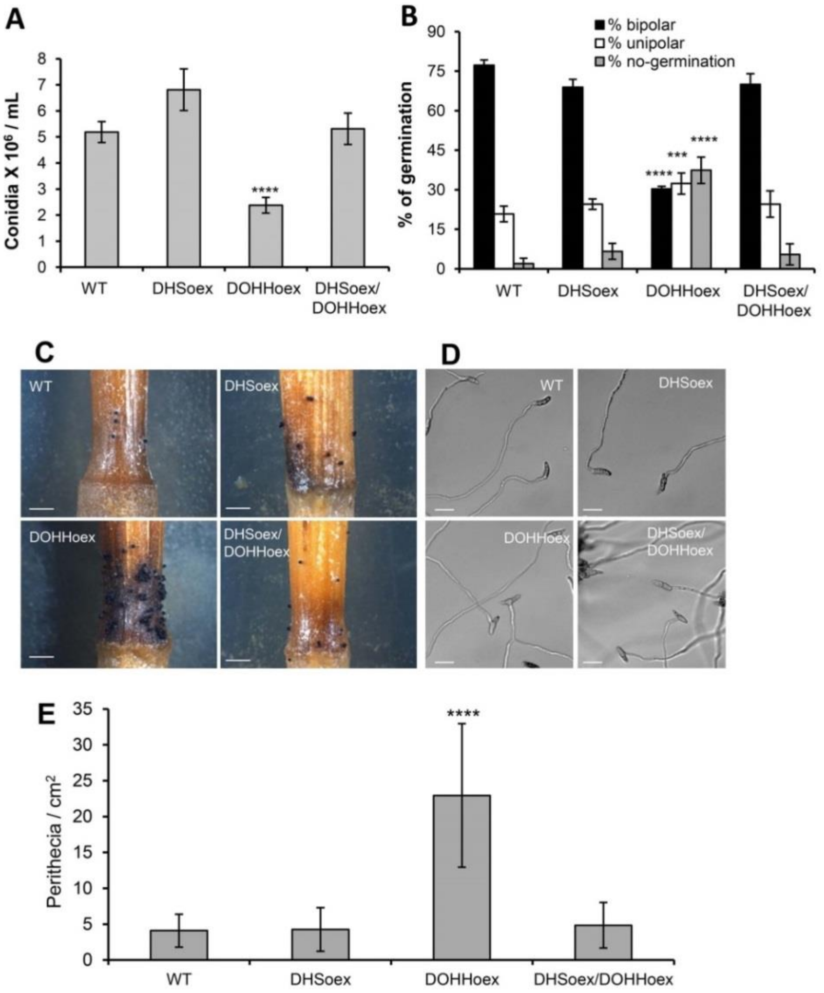

Supplementary Fig. S5 *DHS* and *DOHH* overexpression impair asexual and sexual conidia production and germination. WT strain and DHSoex/DOHHoex mutant produce similar amount of asexual conidia and perithecia. DHSoex mutant produces more asexual conidia and similar amount of perithecia, while the DOHHoex mutant produces less conidia, but more perithecia than the wild type strain. Whereas germination numbers and patterns in the DHSoex and double mutants resemble the wild type strain, the DOHHoex mutant shows a higher percentage of unipolar and no-germinated conidia and considerably less bipolar germination on asexual conidia. Ascospores from every strain were viable. **(A)** Conidia production. **(B)** Asexual conidia germination was assessed for bipolar, unipolar or no-germination. Error bars indicate standard deviations calculated from data per triplicate samples and are representative of three independent experiments. **(C)** Perithecia production on wheat nodes, scale bar = 2mm. **(D)** Ascospores viability, scale bar= 20µm. **(E)** Number of perithecia per cm^2 on wheat node were counted and plotted. Error bars indicate ± SD calculated from data of ten nodes,

two independent experiments. Significance with respect to the wild type: *** p<0.001, **** p<0.0001 (calculated with ANOVA-Bonferroni-Holm).

Supplementary Fig. S6

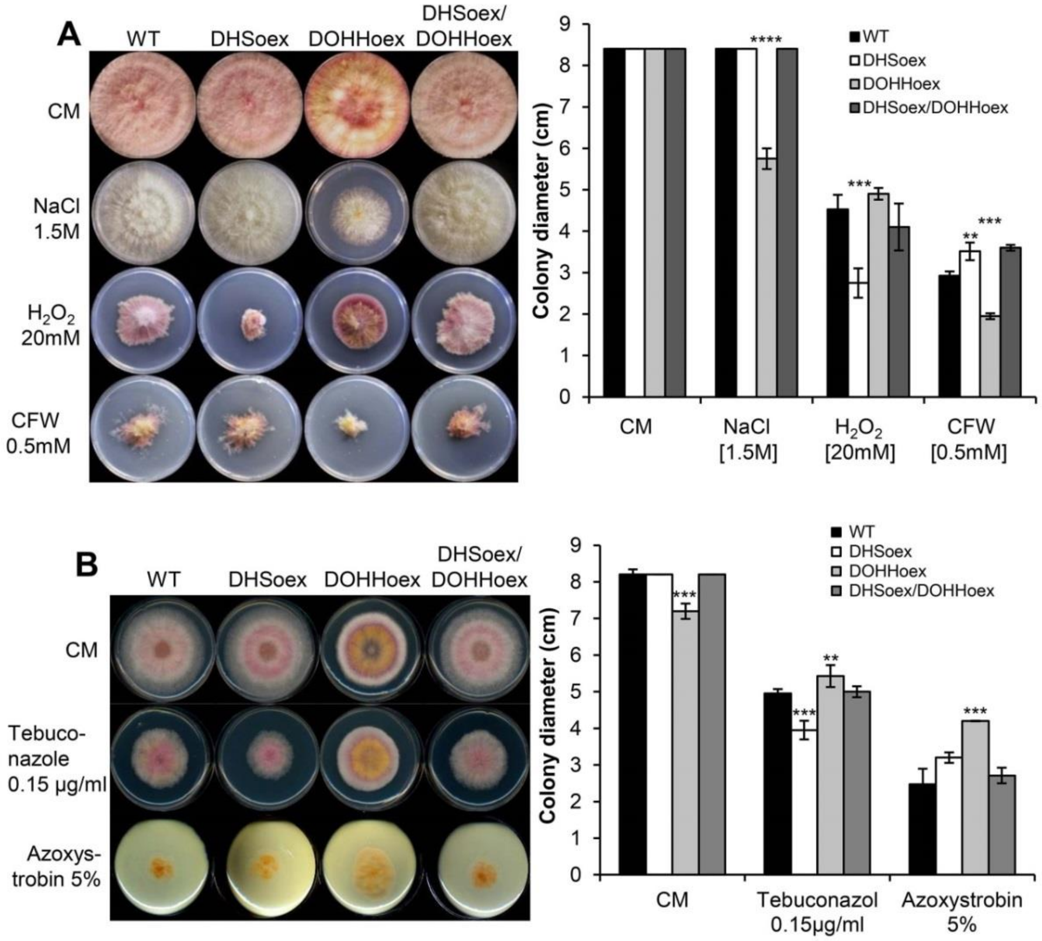

Supplementary Fig. S6 Overexpressing mutants responsible for eIF5A hypusination are impaired in stress responses. The wild type strain and the overexpressing mutants were grown on CM plates supplemented with different stress components. **(A)** 0.5 mm diameter agar blocks of 3 days old mycelia where placed in the centre of plates containing 1.5 M NaCl (osmotic stress), 20 mM H_2O_2 (oxydative stress) or 0.5 mM calcofluor white (cell wall stress) and incubated at 28°C for 5 days. **(B)** 500 conidia were placed in the centre of plates containing the fungicides 0.15 µg/ml tebuconazole and 5% axozystrobin and incubated at 28°C for 4 days. Data shown represent the means ± SD of three independent experiments and three replicates. Significance with respect to the wild type: ** p<0.01, *** p<0.001, **** p<0.0001 (calculated with ANOVA-Bonferroni-Holm).

Supplementary Fig. S7

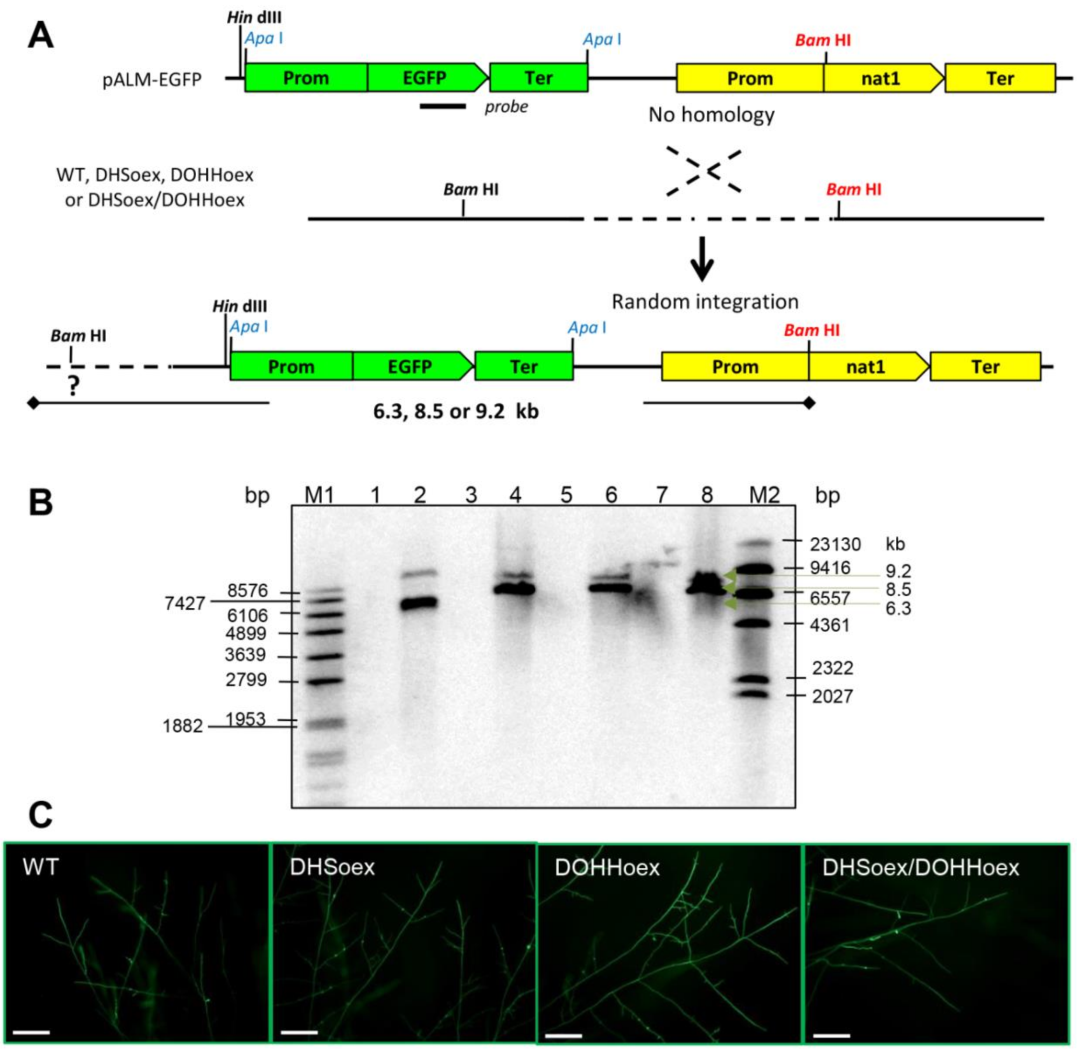

Supplementary Fig. S7 Construction and introduction of EGFP into the overexpressing mutants. (A) The pALM-EGFP vector was constructed by releasing the EGFP fragment with *Apa* I from the pIGPAPA vector. The resulting fragment was introduced into the pNRI vector containing the nourseothricin resistance cassette. The pALM-EGFP vector was linearized with *Hind* III and introduced into the overexpressing mutants by random integration. **(B)** Southern blot show multiple random integrations of the constitutive EGFP. *Bam* HI restriction enzyme was used to digest gDNA from the wild type and overexpressing mutants. EGFP fragment was used as a probe. Lanes, M1: DNA molecular weight marker VII; 1: WT, 2: WT-EGFP (previously prepared with the pIGPAPA vector), 3: DHSoex, 4: DHSoex-EGFP, 5: DOHHoex, 6: DOHHoex-EGFP, 7: DHSoex/DOHHoex, 8: DHSoex/DOHHoex-EGFP, M2: DNA molecular weight marker II, DIG-labeled. **(C)** Mycelia of the overexpressing mutants containing constitutive EGFP grown on SNA media for 48 h at 28°C show no difference in morphology or branching patterns. Scale bar = 0.5 mm

Supplementary Fig. S8

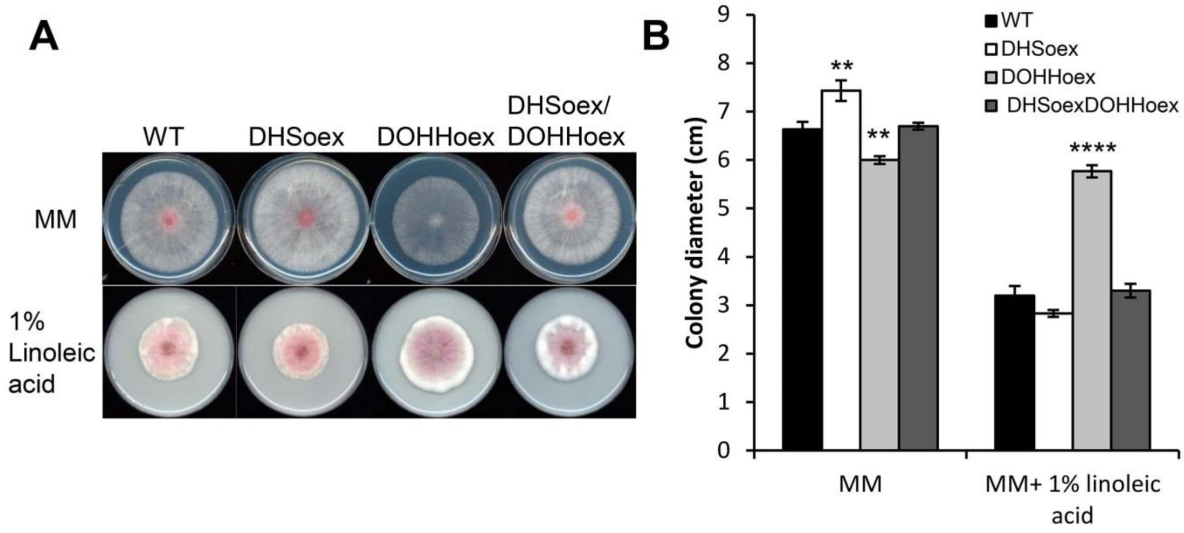

Supplementary Fig. S8 Linoleic acid promotes growth on the DOHHoex mutant. (A) 500 conidia of the wild type strain or the overexpressing mutants were placed in the centre of minimal media plates supplemented with 1% linoleic acid as a carbon source, and incubated at 28°C for 5 days. **(B)** The diameter of the colonies was measured. Data shown represent the means ± SD of three independent experiments. Significance with respect to the wild type: ** $p<0.01$, **** $p<0.0001$ (calculated with ANOVA-Bonferroni-Holm).

Supplementary Fig. S9

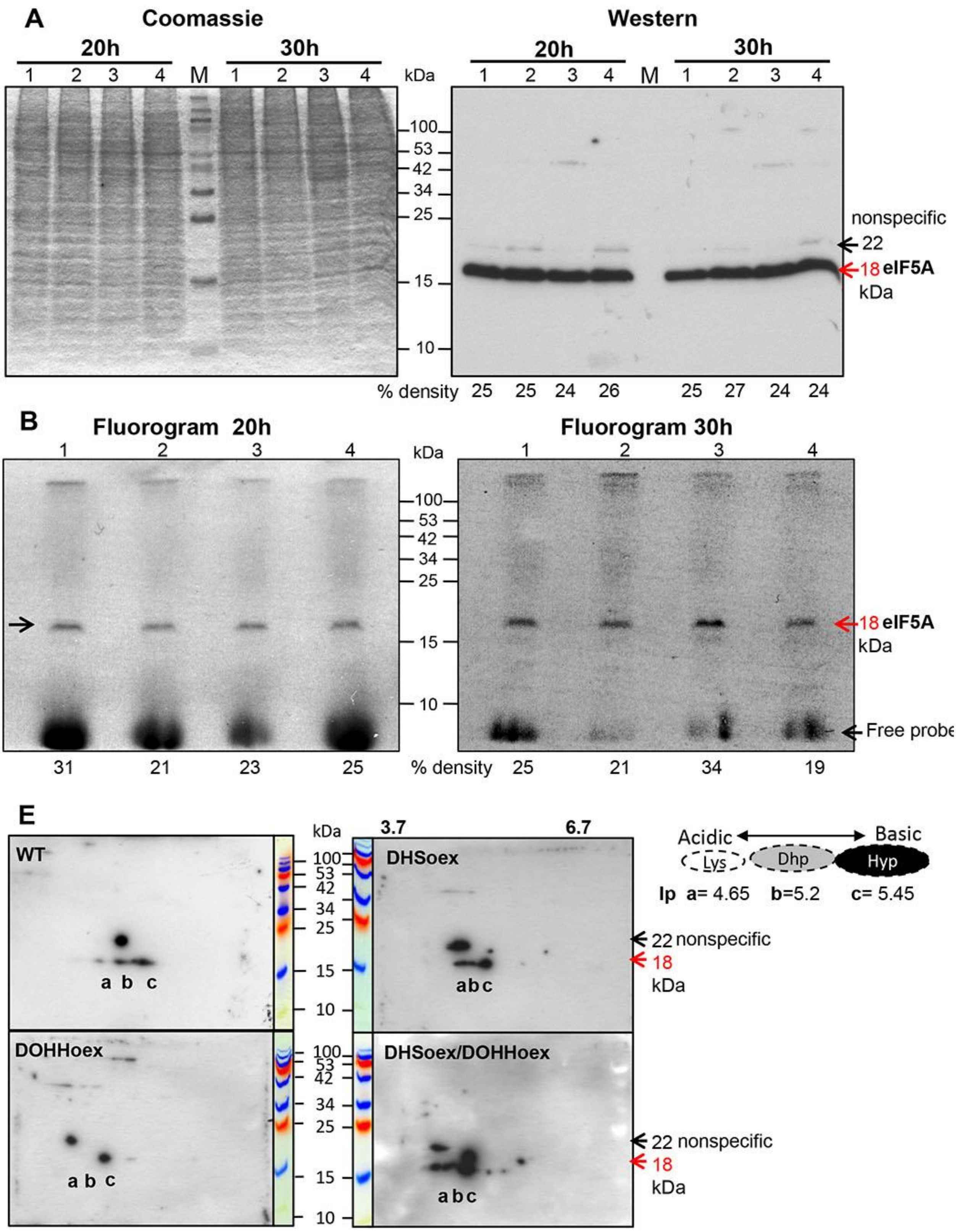

Supplementary Fig. S9 Complete Western blots and fluorograms from Figure 6. (A) 15% SDS-PAGE stained with coomassie blue (left). Western blots with anti-eIF5A antibody detected similar amounts of total eIF5A protein produced in wild type and overexpressing mutants after 20 h or 30 h of growth in CM (right). Lanes**,** M: protein markers; 1: wild type strain; 2: DHSoex mutant; 3: DOHHoex

mutant; 4: DHSoex/DOHHoex mutant. **(B)** Complete fluorograms**.** Wild type and overexpressing mutants incorporated similar levels of radiolabelled hypusine after 20 h of growth in CM (left). However, DOHHoex mutant incorporated more spermidine after 30 h of growth (right). Lanes**,** 1: wild type strain; 2: DHSoex mutant; 3: DOHHoex mutant; 4: DHSoex/DOHHoex mutant. **(E)** The hypusination state of eIF5A in the overexpressing mutants was determined by 2D gel electrophoresis and subsequent Western blots using eIF5A antibody. The three isoforms of eIF5A, inactive (a = Lys), intermediate deoxyhypusine (b = Dhp) and active hypusine (c = Hyp) are detected in the wild type, DHSoex and DHSoex/DOHHoex strains, while the DOHHoex mutant contains only the fully hypusinated (active) form of eIF5A.

Supplementary Table S1. Plasmids used or generated in this work.

**Name**	**Use**	**References**
pJET1.2 blunt Cloning Vector	General cloning	Thermo Scientific, Germany
pCWHyg-PgpdA	Overexpression under gpdA promoter and TrpC terminator from *Aspergillus nidulans* with Hygromicin (Hyg) selectable marker	Wasmann, C. & Van Etten, H. D. (54)
pII99-Pgpd1	Overexpression under gpd1 promoter and trpC terminator *from Cochliobolus hetrostophus* with geneticin (NptII) selectable marker	Namiki, F. *et al*. 2001 (55)
pIG-PAPA	eGFP constitutive	Lee, J., *et al*. 2003 (56)
pNRI	Nurseothricin (Nat1) selectable marker	Malonek, S. *et al*. 2004 (57)
pMW-DOHH-Hyg	DOHH deletion	This study
pMW-DHS	DHS overexpression	This study
pMW-DOHH	DOHH overexpression	This study
pALM-eGFP	eGFP with Nurseothricin selectable marker	This study

Supplementary Table S2. Growth of wild type and overexpressing mutants under different media conditions.

Condition	WT	DHSoex	DOHHoex	DHSoex/ DOHHoex
CM-28°C	+++	+++	+++	+++
CM-4°C	**+**	**+**	**-**	**+**
CM-8°C	+	+	+	+
CM-30°C	++	++	++	++
CM-32°C	++	++	++	++
CM+Congo Red 0.7 M	+++	+++	+++	+++
CM+Sorbitol 2 M	+++	+++	+++	+++
MM+Glc 1%	+++	+++	+++	+++
MM+ maltose 1%	+++	+++	+++	+++
MM+ starch 1%	+++	+++	+++	+++
MM+ glycogen 1%	+++	+++	+++	+++
MM+ linoleic acid 1%	**++**	**++**	**+++**	**++**
MM+ oliv oil 1%	+++	+++	+++	+++
MM+ wheat germ oil 1%	+++	+++	+++	+++
Growth 4dpi on CM, 5dpi in minimal media and 7dpi in cold temperature. **Key:** +++ = extensive hyphal growth; ++ = reduced growth; + = very poor growth; - = no growth/sparse mycelium				

Supplementary Table S3. Oligonucleotides used in this work.

Primer	Sequence (5´- 3´)	Application
FgDHS-ORF-F	GGCCTCCAACTCTGATGCTC	cloning gDNA and cDNA of *EIF5A*, *DHS* and *DOHH*
FgDHS-ORF-R	GATCCTCCTTCCCGATGTCC	
FgDOHH-ORF-F	ATGTCGCCTTCTGCTGATAC	
FgDOHH-ORF-R	TCATGCAGCAGCTCCTGCG	
FgEIF5A-ORF-F	ATGTCCGCCCCCAACGAC	
FgEIF5A-ORF-R	TTAGCCCTGGTTAGGAGCCT	
FgDOHH-KO-F	ATGGTCAGCC CGTGAGCTT	*DOHH* deletion construct
FgDOHH-KO-R	GACCCTTTGGTGCTGAAGCA	
FgDOHH-F	CTAGACGTAATACTCGACGATGC	
FgDOHH-R	CTGAACTGACTTTCAGGCACAC	
M13-F	GTAAAACGACGGCCAG	sequencing of *EIF5A*, *DHS* and *DOHH*
M13-R	CAGGAAACAGCTATGAC	
FgDHS-Bam-F	AATggatccATGGCCTCCAACTCTGAT	*DHS* and *DOHH* over-expression constructs
FgDHS-Bam-R	AATggatccTTATTTTTGATCCTCCTTCC	
FgDOHH-SacI-F	AATgagctcGATACCCAAAATGTCGC	
FgDOHH-R	CATAAATCATGCAGCAGCTCCT	
FgDHS-F	TAAACGATGAGCGATCCGTC	*DHS* Southern probe size 310 bp.
FgDHS-R	CCAGCATCACTACCGTCGAA	
FgDOHH-F	AAGCTCATGGACACAAACGC	*DOHH* Southern Probe size 410 bp
FgDOHH-R	CAAAGCATACTCAGCGTCCT	
11F-GFP	GACCACCTTCACCTACGGC	eGFP Southern probe size 374 bp
12R-GFP	TGTTCTGCTGGTAGTGGTCG	
qF -FgTUB	TGTCGACGACCAGTTCTCAGC	Expression analysis

qR-FgTUB	CGATGTCGGCGTCTTGGTAT	
qF-FgUBI	CTTCACTACACGCATCTACC	
qR-FgUBI	GACAGAAGAACTTTAGAGATGG	
q7F-FgEIF5A	CTGGCAAGAAGTACGAGGAT	
q8R -FgEIF5A	GCCCATAGAGGTGAGGACAAT	
q4F-FgDHS	GGAGCTGGACTTCAACAAAC	
q5R-FgDHS	GACCCGACGAGATGAGATTG	
q4F-FgDOHH	TGCGAGATTGCCATTGAGCG	
q5R-FgDOHH	GCGGTAACGGGAGAAGAG	Expression analysis
qTRI4-F	GTACCGTTATCCTGTTTGCTG	
qTRI4-R	CAAAGGCCATAGTGTATCCGA	
qTRI5-F	ATGACTACCCTCAATTCCTTCGT	
qTRI5-R	GAACTTCTTGGCGTCCTCTG	
qTRI6-F	AGACTTTGTACTCCGAAGAACCA	
qTRI6-R	TTGTCCTTCCTTGTCTTGCCA	
**F**: forward primer; **R**: reverse primer. **Lowercase**: recognition sites for restriction enzymes.		

Supplementary Table S4. Comparison of gene stability of two housekeeping genes for internal control in RT-qPCR.

Method	*TUB*	*UBI*	References
Delta CT	0.701	0.701	Silver N., Best S., Jiang J. & Thein S. L. Selection of housekeeping genes for gene expression studies in human reticulocytes using real-time PCR. *BMC molecular biology*. **7**, 33 (2006).
BestKeeper	1.562	1.564	Pfaffl M. W., Tichopad A., Prgomet C. & Neuvians T. P. Determination of stable housekeeping genes, differentially regulated target genes and sample integrity: BestKeeper-Excel-based tool using pair-wise correlations. *Biotechnology letters.* **26**, 509-515 (2004).
normFinder	0.351	0.351	Andersen C.L., Jensen J.L. & Orntoft T.F. Normalization of real-time quantitative reverse transcription-PCR data: a model-based variance estimation approach to identify genes suited for normalization, applied to bladder and colon cancer data sets. *Cancer research*. **64**, 5245-5250 (2004).
Genorm	0.701	0.701	Vandesompele J., De Preter K., Pattyn F., Poppe B., *et al*., Accurate normalization of real-time quantitative RT-PCR data by geometric averaging of multiple internal control genes. *Genome biology*. 3, RESEARCH 0034, (2002).

Supplementary Table S5. Statistics for *TUB* and *UBI* genes used as internal control based on their values of CP and results provided by software BestKeeper.

CP data of housekeeping Genes by BEST KEEPER		
	TUB	***UBI***
n	36	36
geo Mean [CP]	22.31	21.26
AR Mean [CP]	22.38	21.34
min [CP]	20.04	18.70
max [CP]	25.16	25.68
std dev [+/- CP]	1.56	1.56
CV [% CP]	6.98	7.33
min [x-fold]	-4.83	-5.89
max [x-fold]	7.20	21.44
std dev [+/- x-fold]	2.95	2.96

Supplementary Material and Methods

Materials. Restriction enzymes were obtained from Fermentas-Thermo Scientific (Germany) and Promega (Germany). Custom gene-specific oligonucleotides were synthesized by Eurofins MWG Operon (Ebersberg, Germany). Nitroblue tetrazolium chloride (NBT) was purchased from SERVAelectrophoresis GmbH (Heidelberg, Germany). Amplex® Red Hydrogen Peroxide/Peroxidase Assay Kit for measuring H_2O_2 was purchased from Invitrogen (Darmstadt, Germany). RIDASCREEN®

DON kit was purchased from R-Biopharm AG (Darmstadt, Germany). [^{3}H] Spermidine Trihydrochloride labelled in the diaminobutane moiety was from Perkin Elmer (Hamburg, Germany) [Terminal Methylenes-3H(N)]-Spermidine Trihydrochloride, 5mCi (185MBq), product number: NET522005MC. IEF blue strips 4-7 (7cm) and related buffers and reagents were obtained from SERVA electrophoresis GmbH (Heidelberg, Germany). IEF blue strips 5.5-6.8 (7cm) and related buffers and reagents were obtained from Bio-RAD (Munich, Germany). eIF5A1 antibody (GTX111013-rabbit anti-human eIF5A1) was purchased from Acris-antibodies GmbH (Herford, Germany). Anti-rabbit IgG, HRP-linked antibody and Lumiglo were obtained from New England Biolabs GmbH (Frankfurt am Main, Germany). DHS antibody (H-300: sc-67161, anti-human DHS) was purchased from Santa Cruz Biotechnology, Inc. (Heidelberg, Germany). DOHH peptide and antibody (anti-*F. graminearum* DOHH) were produced by Davids Biotechnologie GmbH (Regensburg, Germany).

Fungal strains and culture conditions. The fungal isolate *F. graminearum* 8/1 wild type was used to produce the overexpressing mutants described in this study (51). The wild type and mutant strains were grown, cultured and transformed as described before (16). Minimal media (MM) and complete media (CM) were prepared according to (19), and were used as liquid media or solid media with 2% agar. Macroconidia of the wild type and mutant strains were collected after 10 to 12 days on SNA agar plates, prepared according to (20) in 5 ml of sterile deionised water in a Falcon tube. Conidia were recovered by centrifugation at 4000 rpm for 10 min at 4°C, and the pellet was resuspended in sterile deionised water to a final concentration of 10^6 conidia per ml. Macroconidia of all strains were stored as aqueous suspensions at -70°C. For biological assays, cultures were prepared with a conidial suspension of $1X10^5$ conidia per ml in a 150 ml flask containing 20 ml liquid media (complete, minimal or SNA media) for the indicated time on a rotary shaker at 150 rpm and 28°C. The mycelium was then harvested by filtration and frozen in liquid nitrogen and stored at -70°C until use. For plate assays an agar block of 5 mm was placed in the centre of the plate or a 5µl droplet containing 10^3 conidia was placed on the centre of the plates. Mycelia were allowed to grow for 3 to 5 days at 28°C in the dark until analysis.

Plant material and growth conditions. Wheat plants (*Triticum aestivum* L.*)* from the susceptible spring cultivar Nandu (Lochow-Petkus, Bergen-Wohlde, Germany) and the semi-resistant spring cultivar Melissos (Strube GmbH, Germany, FHB resistance category 3) were grown in a greenhouse with a photoperiod of 16h light at 18-20°C and 60% relative humidity. Maize plants (*Zea maize* L.) from

the inbred line A188 (22) were grown in a greenhouse with a natural daily photoperiod (or additional artificial light when required), at 26-30°C and 70-85% relative humidity.

Constitutive expression of EGFP in the overexpressing mutants and wild type strain. To construct the pALM-EGFP vector, a fragment containing EGFP was excised from the plasmid pIGPAPA with *Apa* I restriction enzyme and cloned into the plasmid pNRI containing the *nat1* gene encoding the nourseothricin acetyltransferase cassette, which confers resistance to the nurseothricin antibiotic (Supplementary Table S1). The pALM-EGFP plasmid was linearized with *Hind* III restriction enzyme and used to transform the overexpressing mutants. The wild type strain containing the EGFP was produced previously (Supplementary Fig. S7).

Expression analysis. For in culture analysis of *EIF5A, DHS*, *DOHH* gene expression, mycelia grown in 20 ml of complete media with a suspension of $5X10^5$ conidia per ml at 28°C on a rotary shaker were used to extract RNA using peqGOLD TriFast (PEQLAB Biotechnologie GmbH, Erlangen, Germany) following the manufacturer´s instructions. For analysis of *TRI4*, *TRI5* and *TRI6* gene expression in culture, inductive conditions as described for DON measurements were used. For expression analysis in plants, wheat heads were infected as described before and RNA was extracted from the infected florets using peqGOLD TriFast reagent. For preparation of cDNA the Revert-Aid H minus first-strand cDNA synthesis kit (Fermentas-Thermo Scientific, Germany) was used. Quantitative RT-PCR was performed using the LightCycler 480 SYBR Green I Master (Roche Diagnostics GmbH Mannheim, Germany) according to the manufacturer´s instructions. Primers used for RT-qPCR are listed in Supplementary Table S3. Relative gene expression was calculated using the comparative cycle threshold method with the LC 480 software for the light Cycler 480.

Perithecia formation on wheat nodes. Dried and double-sterilized wheat nodes were placed on water agar plates and inoculated with a drop of 5 µl of a conidial suspension containing $1X10^5$ conidia per ml. Inoculated wheat nodes were incubated in a growth chamber with white and UV-light and 16 h light photoperiod at 24°C for 3 weeks. Representative pictures of wheat node with perithecia were taken with a MZFLIII microscope (Leica Microsystems, Switzerland). Discharged ascospores were re-suspended in 500 µl of water and incubated for 24h at 28°C with 150 rpm. Microscopy pictures of the germinated ascospores were taken with a Zeiss Axio Imager.Z1 microscope. The number of perithecia per cm^2 from ten nodes each strain were counted and plotted.
